# Supplementary material for: Exploring barriers of household contact screening of index case contacts of pulmonary tuberculosis cases in Sekela district, Amhara region, Ethiopia: 2023; descriptive qualitative study
Source: PLoS One. 2025 Dec 30;20(12):e0339992. doi: 10.1371/journal.pone.0339992 (PMC12753078; doi:10.1371/journal.pone.0339992)
Supplement: S1 File — (ZIP) [file pone.0339992.s001.zip › translated.docx]

Coding

Coding is conducted from the translated data with different colour

**EX 1**

Interviewer: What has been done to these 8 contact families?

Extension 1; Together with their families; First of all, nothing was done to them with their families. Except for monitoring these patients to take only the medication; they have not been examined together with their families because, first there is no support from the district. When the district and health center experts come to support the other, even if they come once or twice a year, they pay attention for another job. They never acknowledged us the need to diagnose the problem with TB patients. We have not received any training before, so we did not have their families examined. Besides, it is said that there is a lack of resources. Sometimes now we just learned; Family, When we learned before, it is said that if a person is a patient of pulmonary TB, it is the family that should be tested. Therefore, sometimes; it is said that there is a lack of resources at the time when these people are sent to be examined. There is a lack of man power. There is a lack of resources. At the time when contacts are going to be referred to health institution; they complain because there is no quick service, so we don't refer them. It is said that there is a shortage of reagents, and we don't refer them that much because there is a shortage of reagents. When they go to the health center, they don't need to be referred because they say we **have the symptoms** but they say, you don’t have TB, so they don't want to go. These two people; People who were found went to Bure Hospital where they were found. They came with the anti TB medicine and were sent to the health center. From the health center to the health post, the medicine came. We gave them two months ago. From the health post they have been taking the medicine every month for three months now, but their families have not been screened. Because there is a shortage of manpower, when we collect a suspect families and call the health center, they said our laboratories are small; we will not send to him, come hither; they say it is. No investigation has been conducted yet. They say that there is another lack of budget for transport. Now, first of all, the distance between the health center and their home is too far to do the screening. Because of the lack of budget for transport, it is very difficult to do the examination because of the lack of transport. It is very difficult for them to go and do the investigation because there is a problem with transportation. We have a lot of difficulty in doing the investigation. It means that there is a problem with the budget and transportation. And it is for other work, but they do not pay much attention to the TB program. The same thing is to be with them in the future and create awareness. The other thing from the **perspective of the patients and their families** is that there is a problem of awareness. There is a lack of awareness. Actually we did not make them aware of it, even if they were made aware of it; the problem is that they are non-committed to do it. They give priority to their work. The patients do not think that the TB disease from them will be transmitted to their families. Even, now they are doing their own work; priorily taking medicine, because it doesn't put them to sleep. And leaving them here because they see their work; Because of their non-commitment and motivation, they don't care much about TB. And those people now are distant; they find it very difficult to bring their families and get tested for TB. The first transport motor cycle. Most of the time, the motors are very expensive, so instead of the farmer spending money; It would be easier if something else was called grain collection. And it would be possible if one contact paid for that motor to come and be screening. It is far away because it is very difficult for the whole family to pay for the motor to come and do the investigated. It has a mountain. And they create reason. And because there is a distance to investigate, they don't want to. So they don't care to come and investigate. Other, neighbors discriminate them; they don't want to expose the disease because they think that. The reason is that if the whole family is examined, something else will happen; Inferior; they think that the society. So they are now left alone; to keep the secret; But if every family who wants to be investigated, the society will isolate us; they think because they are TB patients. Their families will transmit it on to us; because they say that they separate us because they think that; and they want to be kept secret. That's why they don't want to investigate. Some of them have trouble going to the health center, so they had a lot of trouble getting tested before. The transportation is a big problem, when we are asking them to come and do the screening here. We will ask him further

​Probe: You have explained to me that it is a problem of transportation and distance, maybe you have made an effort to go to the people's house and investigate?

Extension 1: We have not made any effort to go and examine their families. Because it is far from our health post to reach people's homes, there is no transportation. There is only motor for transportation. The motor is difficult and the price is very expensive. Apart from that, the road is not convenient. Therefore, we did not make any effort; we did not do any investigation for them.

Probe; There is Lack of manpower and resources. Do you have anything to ask the health center?

Extension 1; There is Lack of human resources and resources at the health center; Whenever we meet, when we meet; When we have a meeting with the health center, we have raise the question because the community will raise a complaint; There is no input when we go there. We always raise the question because there is no one and they say wait in line. They say we will fix it. We will always fix it later, and the input will come.

Probe; In order to provide awareness, they may not be educated or read, so even if there is a distance, could you please explain to me if there is anything that you have given health education on your part, maybe the first work of health extension is going to the church or going to the place nearby?

Extension 1: Now we are actually doing health extension work first to give health education. Awareness is created. But mainly because the district and the health center did not pay attention to the creation of awareness, we told them that we did not pay attention to them because the health center did not pay attention to them. Because we tell them that if it is pulmonary TB, it is transmitted from one person to another through breathing. Always. And we didn't pay attention to the fact that they should be investigated. We create the same impression.

Probe; if you have another idea to add or something that is not clear from the question, raise it

Extension 1: The context in which the question was asked is clear. But what I am giving you now is because I think that your university (the project) will prepare this question for the future. So, collect this worm now because it will lead to something good, so that it doesn't just stay here, tomorrow TB patients will be transmitted from person to person.

Interviewer: Summarizing what has happened so far, please explain from the perspective of the district that support will be provided, but they have told me that no attention will be paid to TB. They told me that there is a lack of resources and a lack of human resources, for example, there is a problem with laboratory reagents and service at the health center. From the point of view of patients, there is a cognitive problem, putting their work first and not paying attention to the disease. In terms of the community or the area, they have revealed to me that there is a problem with the society's attitude so far, that they isolate patients and that patients face transportation problems due to the distance with their families.

EX 1; I don't have that much idea. Thank you for paying attention to the TB program. We will also do good things in the future.

**Coding of EX 2**

Questioner: Tell me about your experience, what are the obstacles to health experts investigating the TB status of people who live in the same house with a TB patient?

X2: Thank you. As she explained, the same is true from the perspective of the health system or the government and from the social and cultural perspective of the health professionals. The first is the lack of support. Lack of support means, for example, the lack of connection between health centers and health centers. Apart from that, lack of training, as I am a health extension worker, there is lack of knowledge about the nature of TB. **Mainly from the government's point of view**. There is also lack of resources. When we say lack of resources, we mean the absence of reagents or other things. Apart from that, when we go and send them quickly, they are not handled quickly. Other than that, not giving feedback. Not identified as This is a weakness, this is a strong point not given feedback; considered as a gap from the point of view of the government.. These are listed from the government's point of view. It means that we see them as creating the gap. Apart from that, there is a shortage of human resources. For example, because I am one, entering from different departments and not paying particular attention to one work, for example, it means the one like TB. Apart from that, there is no transportation budget. There is a transportation problem when we want to go and bring our TB patients. This is one of the gaps. These are barriers listed from the government's point of view; we see them as creating the gap. And then when we go from the point of view of health professionals, the lack of knowledge about the TB patient's family screening. The lack of knowledge, because the health professional did not have trained to know whether the families of TB patients have TB status or not and screen them. For example, we know that it is the patient who is investigated, but not have awareness about contact investigation, because there is a gap in training for the family to be examined, because there is a lack of knowledge, we are unable to do it. Besides, there is also a commitment problem related to not taking training. Commitment problem mean not planning, I can do this work by myself not interested. Apart from that, there is work over load, working in different department. For example In terms of my work as a single health extension worker, there is a lot of work, and I have to go to different departments like under five, vaccination and maternity. It created as an opening. Because one person from different departments from mothers to children. When the work load is heavy, there is something to put that work behind. For example, TB. So this is one of the gaps. It is because of the lack of manpower that I mentioned earlier. It means that it is the main gap. We explained earlier that we do not do a TB screening for TB patients contact, because we care about the patient, but because there is a lack of knowledge, we did not do it for the family. We shall screen contacts. But we did not screened them, because lack of awareness of health professional. The other, when we see from the **point of contacts and the index case,** there is lack of awareness, and we didn’t create awareness for them. First of all, they are not committed to be screened, give priority to their work. It means they don't want to go health institutions and be screened, give priority to their work. Apart from that, when we start from **social and cultural aspects**, there is distance t from home to health institution. As I mentioned earlier, I mentioned that there is a problem with transportation. Even if they travel three hours is more than that. There is a river. So this is a challenging thing. When we start socializing, people are discriminated on social; when there is a pulmonary TB patient in a family, the society discriminate them. As I said before contacts and the index cases are discriminated from social activities like edir and senbete. Because as I mentioned earlier, instead of their health, they focus on their work. From the economic view I mentioned before that there distant and difficult for motor transportation, there is lack of money for transport or very expensive **in low economic** status. In short, this is what I have; not explained much in terms of health extension knowledge. Thank you.

Questioner: What is preventing you from going and investigating?

Extension 2; as I tried to explain earlier, TB patients are not forced to come as you said. We need to go and save them where they are. There is also a no transportation budget. Apart from that, there is also a gap in our understanding.

Probe; what kind of support does the government give you?

Extension 2; I tried to explain it earlier. From the point of view of the government, this is about leaving TB disease behind, giving priority to mothers and children. It means that there is something to put it behind, to see one activity as better than another. When we see it from the government, there is a lack of supervision and control. Because we have prioritized children and mothers, not TB.

Questioner: What is the relationship between you and the health center?

Extension 2; as I tried to explain earlier, the connection between the health center and health post is loose. Because TB is a forgotten activity. There is no way to make the patient live in a family so that the family can be seen quickly and the patient can be treated quickly. Because there is something, our bond is loose.

Probing; what is your impression that the family should be careful about being tested? What did you do?

Extension 2: Nothing we did. As I tried to explain earlier; we have not created awareness. I explained to you that there is a knowledge gap. Even if a patient comes and is diagnosed with a disease, we do not have the ability to explain that he will be treated because there is a skill gap.

Probing; it is said that there is something to go to traditional medicine from a social and cultural point of view. How is this?

Extension 2; Sometimes this did not give us the lesson. I have tried to express it in every way. There is something about the disease sometimes pushing the disease into the culture. And they believe it is home sickness, because there is something they say, the society is the same: it is used by the culture most of the time.

Questioner. Summary...if you're going to finish it at the end?

Extension 2: The same things that are set as a challenge: I believe them, as I tried to explain before, and the problems are still getting worse, believing that culture is a home sickness, having a gap in awareness, I mean to say that this is the main problem. My society does not recognize this. There is a wide gap in the fact that an institution cannot be treated and cured, and that we do not create awareness, and that the society does not know. As the problem is getting worse, there are things that I have mentioned from the government's point of view, there are also things that I have mentioned from the professional's point of view, and from the social and economic point of view, the problems that need to be solved are the recognition of the professional, and if the society does not recognize it, the problems will get worse.

Questioner: Thank you too.

**EX 3 coding**

X 3; what is the problem? There is a general lack of awareness among the community. In terms of awareness problems and work, they give priority to their work. Since it is a rural area, they give priority to their work. What else is there to give up, if they know there is TB; they will assume I will die tomorrow. They don't pay attention for screening and have no commitment and initiative .there is no other problem. But for the future, we are thinking of paying attention to this in the same evaluation. Another from the health professionals and from the health institution, especially, is not giving feedback, the lack of training, the lack of feedback, and the lack of evaluation and monitoring. Evaluation and monitoring should be minimal. When I say that training is not provided, from the point of view of the **institution and the government**, the lack of sufficient training.the institution should focus on the lack of training from the government's point of view. On the other hand, when there is TB case only follow that case but don’t care for the contacts whether he transfer or not. Because training was not given properly. Then the evaluation and monitoring itself was done every three months and every quarter. But the evaluation and monitoring is to draw attention. There is **no meeting** to evaluate the work of TB. Feedback used to be given by us, but now no feedback is given. The lack of feedback is the reason why it was not given attention. Another thing is the lack of economy in the community low economic /. They also have problems such as distance t. Now even if they know the reason why they don't come, even remotely; For example, if they have the enough money, they will even go by motorbike. How can they do that ten families, they give up because the ten families always have the same car, how can we go back like this? Especially in terms of ups and downs, I think it is because they have economic problems that they have not paid attention to. Another is discrimination, now, for example, like in our society, where TB is not given much attention, there an isolation. Now if it is said that someone has TB; he is afraid of taking drugs. TB patients come early at night, but there is isolation. They should be isolated. Now they think that if they get TB, they bring some kind of pressure on themselves and say, 'What's wrong? But because they don't know this, they think that they won't be cured. Because they give up hope and let others say that they are the only ones like this. Therefore, we identified this assessment because they have problems of isolation. So, it seems that we should pay attention to it in the future. Another problem from **a health professional's point of view**, a shortage of human power for example, I work alone; there are different vaccines, there is maternal care, there is a package, it will be multiplied every day. It is a bit difficult for us to do everything in order. And there is three-day schedule of vaccination, we always follow the children. In addition, when we give albendazole or vitamin A for stomach worms under the age of five, we give it in large quantities. We have work over load. It's rural, we work. In addition, if the mother's conference does not come, we will say what happened to you. In addition, we choose children who have dropped out. We have a lot of work load to do, because I am alone because I am alone, that's why it is not paid attention to.

Probing: What is the problem in terms of resources and human resources?

X3: In terms of resources, it is impossible to say that we always get the medicine properly. Why is, there is lack of resources from the **government's point of view**. From a professional point of view, the lack of professional staff means that they have a serious problem. I think that the health center also has a staff problem. Apart from following up and treating the sick one by one, they say that they give up the priority and the support from us is less because there is a shortage of a shortage of human power. Especially in terms of our kebele, because I am working alone, there is a shortage of manpower. If the government sees this and monitors us day by day, there are three health extensions for at least some of our kebeles, but I am alone. I think that if the government works well one by one, we will serve the society better. I think it would be better if he followed up on that in terms of input.

Probe: What did you experience when you went and screened?

X3: Yes, we go and talk as much as we can. First, we go and talk and advise, but it is not said health extension shall screen. We have not done it yet. There is no health extension who screen at all. As I told you before, there is a lack of expertise, so they should not follow up and go at least from home. If so, I don't advise them to recommend something better. But we advise them to go with attention. The best thing for the future is that if they miss any transport while resting, they should come slowly one by one, and in less than a week, just go and pay attention and advice them to go and at least go to the health facility to be treated or we'll have them screened.

Probing;. What is wrong with culture? If you explain to me the problem from a cultural point of view that keeps them from investigating?

Extension 3; from a cultural point of view, it is very important to ignore them. Culturally, it is not well addressed. As long as it is rural like us, the surprising thing is that we have these opportunities because we have to work. At this time, thanks be to God, they go with great prayer, and others believe the same thing, they go carefully and do cultural treatment. But when they said it was TB, they didn't pay attention to it. The community didn't wake up. It was just a deadly disease or something that they brought. Especially from one of them, I was told that there is nothing that can be cured when treated.

Interviewer, summary...Finally, if there is anything you would like to add, there is something left over;

X 3: Yes, if I mention this, the truth is this, and it would be better if 100 percent of that was removed, even 50 percent.

**EX 4 coding**

X 4; There are many problems that prevent us from investigating of contacts. Of course, even though we don't know clearly that it will be investigated, we have a slight aware of being screening contacts; there are different problems for us to investigate them clearly. For example, when you see it from the **governmen**t, we must take clear training to investigate contacts. In order to support and advice them; we must know the inside before. We have to wake them up. We can't wake them up or teach them when we are not given training. The society can be investigated in the form we have explained to them, but not just. And the attitude of the government about **TB contacts is not that much**. As you can see, the activities of mothers and children are not equal to the activities of TB. Because mothers are given priority. The woreda governments only said identify if there is TB suspect, but they don’t give training about what is TB and how contacts are screened seriously. Besides, by going to church; we said to the community if you have a cough, you should be examined, but we believe that there is no health extension, which knows what kind of cough the person has and we don’t tell them clearly due to lack of training. Because we have no aware. Because of that, the government is cold and does not pay attention to it, so we don't get much training. When they say they need to be examined, we tell them that you should go and examined at the health center. But if we tell them that there is this this problem, people go. But since we are not awared enough, we say go and get checked, and they didn't go, they didn't get any check-up. Apart from that, there are some gaps on our side as well as on the professional side. Yes, those gaps on our side are they said, sent to the health center to examine the families of the sick, and since we don't know the problem and the harm, we don't send much contacts and we don't have an active participation to do that. Apart from that, when the contacts go to health center, not only the barrier is from the extension which we sent them by paying attention, but the health workers in the institution don’t paid attention to the ones we sent, they tell them once there once there to do the examination, and they come back by giving up, with no screening when they complain, and this is because of the lack of attention from the experts and the health center. This describes the problem of the professional. Apart from that, it is **not given priority**. These TB patients and contacts are not given priority. Now, any examination of other mothers is done before anyone else. They are given priority to be examined, before others investigate. But it is good if priority is given to those who go as TB patients. In fact, it is good for the health center if they are treated quickly so that they do not spread it to others. Because it is like that and it is not possible to do that, the person who is seen and treated is different. Because when you look at the number of contacts and patients, even from us, as I told you now, there is only one patient. But if it is examined correctly, there will be many patients. If it is given attention and treated, there will be many patients. From the point of view of the government, in addition to not providing training, one of the problems is not **evaluating** the work done, not giving feedback on failed tasks, not giving feedback on the existing things that have been done or not. There is something to say that there is no such thing. Another thing is that in terms of resources, we don't have any form and anything for identified contacts people and come back with nothing done. There are things that need to be done in terms of resources. The other is lack of a focal person to identify TB suspects alone with no other job is a big challenge. Apart from that, there is a lack of enough laboratory professionals. The shortage of human power to go to home of contacts and take sputum and examine it; these are the problems. Apart from that, there is also the problem of transportation budget to move people from very far places and bring tired people and to go to home of contacts. These things are one of the big reasons for the failure of the work. **Not reviewing** and evaluating the work of TB works regularly. Not evaluating these cases regularly like other cases is a big problem. Another thing I would like to add to the professional is **~~not giving awareness~~** about the families of TB patients. If they had awareness now, if we didn't even check them, contacts will say my sister is sick, my brother is sick with TB and I need to be checked like any other disease, lack of awareness is one of the problems. Apart from that, the lack of commitment and motivation is the biggest challenge. Another, as I explained before from lack of laboratory professionals and ours is not enough health extension workers. I am the only one who works in this health post. Currently, there are more than 18 tasks of extension. There is work over load to maintain the quality and to carry them out. Workload is one of the challenges. Because when there is work load; there will be dripping. They are also a problem because there is always something to take with you and leave something behind. Another thing is that when we go to the patient and contacts, there is lack of awareness that TB disease is transmitted from one person to another and the need of contact screening. Even if they know, not to think that there is something that poses such a threat and this is one of their challenges. Apart from that, they should give priority to their work over screening, to give priority to their work. Lack of active participation in TB diagnosis: lack of motivation. It holds us back. They say cough is a cough that bothers me today, but it will leave me tomorrow, there is something not to think that it will get worse. It is like that. The other is from the point of view of their **social and economy**, and the same as the situation of our country, since they are economically poor, there is lack of money for transportation to come here, and there is no transportation for them to come from the countryside to the city. Since their sputum is examined in the health center, In terms of their low standard of living, this creates a challenge. Apart from that, there is something like discrimination and discrimination of their social life. It is known that it is transmitted from one person to another. But it is also very similar to treating a person who has TB as another disease and ostracizing a person who is said to have TB as something else. Because these things exist, this creates a problem for us. Apart from that, the fact that their homes are very distant, there is a problem in itself to bring all the families for testing. The distance t from the testing place to their homes. It's a problem in itself. Let's finish what I have to say.

Probing; what is the support and feedback given to you by the district or health department about TB patient's family examination?

X 4: We are told that they should be investigated. But they should be investigated, well, just like any other activity, people who have been diagnosed with TB today should be their contacts tested today or they should be tested is We don't know what clearly, we have never been trained. Whether sent or not, is not included in the **review meeting**. It shows that the government is not paying attention.

Probing; will there be an impact on traditional medicine and thought not to be investigated?

X4; said, it has influenced. As you can see, if they are not given the awareness, there is a community that trusts their neighbor rather than the professional. Like our rural community, now if you say TB is transmittable and families shall investigated, that they don’t hear professional, they believe the joke of the community. If we are not given our training, we will not be able to train them. Now, if you have to take a little something knowledge and say the community to get tested, Since Ekele has TB, you don't even get a response when they asks you to why to get tested, If you don't know it well; that’s why it has an impact there are traditional healers there, so there are many patients who use it.

Interviewer; finally, if you have anything to add;

X 4: That is, these things that I have said are things that can make this problem worse. Also, I would like him to provide training to the community and respond to the questions that are being asked. What he has an impact, whether it is on the culture, the government, the professional himself or the community. Because that is the case, a comprehensive program like mothers and children should be given feedback. It is better if it is evaluated regularly. This is all I have.

Interviewer; Thank you.

**Patients coding**

**P1**

Patient 1; 4 month.

Interviewer; 4 months since you know.

Patient 1; yes it’s been 4 months

Interviewer;. How long did you start taking medication after you know you had TB?

Patient 1: I started on my fifth day. I started on the fifth day after I was told that I started on the fifth day. I'm still taking it. It's been 3 months to three weeks now. I'm taking it. They sent me to the health center and I am taking it now as much as possible. They gave me medicine there and sent me to the health center and now they sent me to the health post.

I went to the health center, but they couldn't find TB, I gave them the sputum but they didn't find it. After that I went to Bure Hospital and TB was detected there. They gave me medicine there and sent me to the health center.

Interviewer; Thank you very much. Is it a health center that you are currently following or health post?

Patient 1: I am currently being monitored by the health post. They sent me from the health center to the health post.

Interviewer: At what time do you go and bring the health post?

Patient 1: I bring every month. But the road is very distance t the motor is very expensive. The motor is also very far away. The health center and health post is very far away. Since I am a farmer, we have work priority, so I go and bring it every month.

Interviewer;. Very nice, how many people do you live with now? It mean outside of you.

Patient 1, there are 4 other than me who live with me

Questioner: Have you been examined or asked anything about them by the health post or health center experts?

Patient 1: Not available yet. I didn't know either. We are not having aware of it. We did not know that they will be investigated. They didn't tell us either. When we go, it is said that they are not there. The government should focus on this. We ask it to pay close attention.

Probing: What do you think is the problem with not testing them for this? You said you did not know that they were going to investigate something that you mentioned earlier. Is there anything else?

Patient 1; the distance. it is very far awayt from our home to health institutions. The road is very far. Not to mention the motor, the same motor costs a lot of money. And when we go, it is said that there are not many experts in health extensions. It is said that the working material does not exist. If we go on a holiday, they say there is no working material.

Interviewer: It's good. Mainly, now you see your work as if you are busy.

Patient 1: yes very

Probing;. Not being present when you go on holiday; they said things should be controlled, but what will the community or your neighbors say about you having TB?

Patient 1: Ever since I was diagnosed with TB, there has been social isolation. I feel stigmatized. Isolation appears. It is very common in the neighborhood that I will not go zikr and mahiber. The same father told me not to go. They told him.

Probing: But when you started taking medicine at the health center or went to the health check every month, were you not told to bring your family?

Patient 1; No, no, I was not told that

Questioner; Thank you very much. It means that there is a different problem with you, the distance.

Patient 1: AW

Interviewer;. It seems that almost all of our questions have been answered or added?

Patient 1: The government should do evaluation and monitoring for health workers. Health workers are not available when we go. When we go, they are very, very hard to find. They said that when we go, when I go on a holiday, the road is far and the road is far from the health center. They didn't tell me. When we left, they said they didn't exist. The government said they didn't exist. The government should monitor it.

Interviewer;. Thank you very much for the information you gave me.

**P2**

**Coding of words of patient 2**

Interviewer: Tell me what you have observed about the obstacles to health experts investigating the TB status of people who live in the same house with a pulmonary TB patient.

Patient 2; I thought that the barriers of not examination of close family members of the TB family, as you told me, meant that they should have examined the family together. ………………….. From the point of view of you and your family....socially, economically and culturally....I take it that there are many problems. One is **from the government's** point of view; I think that there is no monitoring and control of health workers often. Why can I say this? Now, I usually take drugs for about a month. The place I come from is far away, with coming by crossing such distant road, the health center and the health posts are closed, they don’t on the working place they absent. Again, as you say now, there are many other people who use TB like me. I see that they are proud like me. Their families were also not investigated and found nothing. Like me,. There is not enough supervision. I don't see anything when they come and supervise them and support them. Now, like teachers, now there is supervision, there is supervision every day, but this thing is loose on them, I think their relationship is loose. Another thing is, I think there is a problem with the **construction of an institution**. If I take it as our region or like ours, there is a health center in the area where I am now. There are also health centers that serve the region. You can see the health center in every kebele and you will be surprised. It doesn't have enough buildings. It does not look like a health center. If you see it being used as a TB room, you will be surprised. It's just something in the field, it's not attractive. It's just a place where medicine is kept. Sometimes, even a bird can come in from this room and ruin it. I doubt it. It doesn't have enough class on him. There is not enough human power. Now, from the point of view of the government, there is not enough man power there is no man power. Now, from the point of view of the government, there is not enough man power. Now, the health worker who is treating me with TB often works another class there by the expert examination other department, when he is examined there, I call him and give me drug. I think it works and he comes and gives it to me. When I barely developed, he would come and give me the medicine himself, and I don't think there was a TB doctor assigned to him on the spot, I think he would work elsewhere and he would come and give it to me. There is a problem with the supply or what they call the test. Many times, they hire me saying that we don't have the working material. Now, for example, now, for example, when I was examined, they even suspected me of TB; now we don't have the test, you can use this drug for pneumonia and you will be tested when the test comes next time. When I was tested, they even suspected TB. After I arrived, I was examined and found that there is a problem. The families of TB patients are not treated quickly. The health professional himself does not come to work on time. There is a shortage of human power there are four professionals working on the building. He said that it is a lot of work load there is also a health extension on the health post. Also, when a laboratory with a symptom is seen, it means from the community's point of view, and when a laboratory is seen with **a sign, it becomes free,** so I was disappointed because it was found in the second or third area. The problem in the area and the society is that when he has the same symptoms, he comes by thinking that he will get TB. There is something of despair and he leaves and even goes to another area. Another problem is that there are many people who are not investigating by going to the traditional medicine area, saying that it is TB, but it is gout. There are many people who have to go there. Another problem is the distance to the institutions. Now, for example, it takes me about 3 hours to reach the health center. It is a problem if I say bring the whole family and examines them. Now there is no transport because the area is far away. Another thing is that, as I told you before, there is a work load for the health professionals. From my point of view, when I told you, bring them to me, I didn't know that a TB health status screening was going to be done. Because we don't think that the disease exists, we often get sick. Bring them all together and examine them. Now there will be a thing of discrimination and isolation. Now, a person with TB who often will be excluded from things like my Sunday, from the association. Sometimes he puts it in, but the engine itself is expensive. If you don't ask for anything, he pays him and brings the whole family to be examined. Because we think that there is stigma and discrimination, most of our society also thinks like this.

Another thing is that, as I told you earlier, there is work load from the point of view of health professionals. From my point of view, then, when they tell me to bring them to my point of view; I did not know that there was a TB health screening;I didn’t know that there was a survey; For example, I didn't know that the whole family should be examined now, that's why they haven't been examined, but if I know about it, I can examine it. Another thing is giving priority to their workload. It's because we have a small number of people to get tested. It's because I don’t have intiation and motivation. It's from this point of view. The other thing is from the **social, cultural and economy** point of view. As I told you before, if you look at it from the perspective of now, if it is said that if TB is found or if I bring my whole family and get tested, now there will be something of stigmatization of a person with TB. He is excluded from things like association. As I told you before, the distance from home takes more than 3 hours, so he is there. From an economic point of view, due to the distance, it is costy and there is lack of money to bring the whole family by car. He says that he should bring him for examination, because we think that there is stigma and discrimination, because most of our society thinks so, after he is diagnosed TB he slowly finishes his own medication. He does not say anything except himself.

This is my idea. If there is anything else I can add

Interviewer; Thank you very much; is there anyone who has come to your house and asked him to come and examine your house as much as possible?

Patient 2: No. Not to mention; as I said before, even if I was sick and go to the institution they don’t give care. One day they say there is no working laboratory technician; once they say there is no electricity; Once again, what is a probe? A diagnostic tool; they tell me that give the sputum, it will mix with it. I myself have been examined by how much they struggled while saying that; and it is not usual for them to come home and check anything; No from our area.

Probing: Oh, from your point of view, what you will do in the future?

Patient 2; Oh, after this; If I know this, I will bring my family to investigate them. Without saying tomorrow today; If the health worker examines voluntarily and if they don’t say As before, there is no electricity; no equipment; Because I will also work place; I will examine them. Not only my family will I tell those who use TB to investigate. I didn't tell them yet, but I will check them out.

Interviewer: maybe; finally, if you have anything to add or anything new? Let's finish

Patient 2; Perhaps I will add here the information that you have given me, but you said that you are working on TB, as you introduced me to, from the introduction and when you finish studying this study, I guess that you will give it to the government or the relevant body; And he thought about it; Like me, if you give your opinion on the construction of such an institution to make the rural community accessible, you should also give your opinion and build your share of institutions. For example, the rural health center operates based only on quantity, but the services provided there are insignificant. There are only health centers that look like hospitals when we go to other places. But the ones in our area are in different rural areas of Ethiopia. I think there will be such a problem. If it is possible to train people well, if one goes out, one should work in two or three. They said that if we pay attention to this disease from me, we should pay attention to it because it is getting less attention, we should also pay attention to the society if they come down and educate them. They said I know two or three people from our area who are sick with TB and until now our society is suffering from this disease. I think it would be good if a teacher supervisor like us from the top to the bottom assigns something and works while being supervised. Thank you.

Interviewer: Thank you very much, teacher, you have given us good information, so this research is not to rest here as much as possible, so what we are doing is that at the end, the results will be sent to the district office, the zonal health department, the regional health office, and the university, and the solution will also be tailored to be implemented. I think that as much as possible, efforts will be made not to be only on paper. In the end, I am very grateful

Patient 2: Thank you too

Interviewer: Thank you very much, has a good day

**P3**

**Coding of words of patient 3**

Patient 3; Well, the problem may be that we live in a rural area. We are not **users of existing media**. It is far from the health post and the health center because it is far away from the existing social life. It has a slope; it has a river; that is one of the main problems. The second is that we don't have the awareness. It's only now that I'm getting the recognition after I started medicine. I think it's a problem with professionals. How can you tell me? They had to come and give recognition at every meeting. It means TB or other diseases, but **~~don’t create awareness~~**. The third, as I told you, is the transport. The last and the most important thing is that even when we come here to the health center or the health center, there is a situation where there is a not quickly serving problem. First, they say there is no equipment. They say there is no manpower. He is out this is how it happened. It is very difficult. It's not like I'm going to tell you now. It doesn't seem easy to tell you because I and that. Now we are farmers, you see, we are human beings and we will be happy if you bring all this to a solution.

Probing; Thank you, you tried to explain to me briefly, perhaps what is the commitment on your part, because of your work and what you give to this issue, maybe there is commitment on your part, and you have made an effort to investigate.

Patient 3: No, doctor, how can anyone say this? What is more important than health? It is less in terms of recognition, but how can we say it is our job, what will we do without health? It comes first

Probing; Maybe it's the reception from the community, maybe it's your neighbors, or other people close to the community, what was their attitude about you after you were told that maybe you have TB?

Patient 3; I of the community; Even you will be known in our society; It means our whole way of life; If something is said, isolation behavior occurs. There is something that keeps you from participating in social life together. I would have wanted to join the association myself. What can I tell you? What can I tell you? Families will be worried. There are many things and after being told that he has TB, I won't even go to the association house. I will not join what is there. If I remain silent, it means that what they say is because they are not recognized in the current situation. Since they don't know, I can say that I am isolated.

Probing;. Perhaps after you go to the health institution, could you explain to me the issue of health professionals being in place and being treated quickly?

Patient 3: Well, the one at the health center is where they gave me first. No need to deal with it quickly; If there are, they will host us. But since ours is far away because we walk a long way, if we spend a little time, they said the time has already passed, come to tommorow. How is our medicine interrupted? We asked, but we have not been given a solution for that; and there is no health worker who going to sit there as an independent professional to work permanently. They just kept it as an extra job. They said he works over night, he had gone to house, wait, there's no key, and there's a bit of boasting.

Probing; what is the problem in related to the government? Maybe there is something to rise from the side of the government?

Patient 3: yes, through the government; First, in order to be the user not only with TB, but also with the others; they shall make a bridge for us,; He paved the roads; To get a Bajaj or a motor; It takes us a lot of time to get into the motor and the Bajaj; And if he does that to us. Second, when we come to a health center or a health center, if an independent professional assign a working professional and manages it within the available budget, my knowledge is that much.

Probing;. Maybe you will add it in the end?

Patient 3; Doctor, if you take it for me, if you are going to pass it on to others, it is okay if I take it not only with TB but also with related infections?

Interviewer; mainly, it is better if it is related to TB.

Patient 3; so, if we don't come directly to the health center, if we ask for the resources nearby and enter the checkpoint, it would be great for us, because we are farmers, we don't come because of our time.

Interviewer; Thank you very much. It would be better if we had enough here for today, thank you

**FOCALS coding**

**F 1**

Focal 1: It is true. Then, as it was before; a total of 15 of the 36 have been screened so far. On the other hand, there are 21 screens to be made; there are some that have not been done even by us. As I mentioned, this is because there are many problems in one way or another; So, there are many difficult things both institutionally and ourselves. So far, the society has not been able to reach each of us. Maybe we recorded them, we will tell them with the health extensions as soon as they show the signs and send them as suspects; I think that the family of Engelen's family sent us the trigger; because their family is sick tomorrow, I think that they should be so obstinate or not so strict like the other activities. We know that these many people will become TB patients tomorrow and that they can spread even more. But also, the one on our side and the one on my side, they support the health center from inside the health center to the health post. It's not that much of a concern. They are in the area of health centers, they pay attention to those who have symptoms or have a cough, but they are still in contact and are concerned. We had to go and reach their homes, so they are still at risk of disease. But everyone should be screened for TB so much that the family or the contact should be screened, is it a concern? Therefore, if everyone is trained in life, when everyone goes out for support in every area, he would be able to see these as concerns and give answers; and the training has the some limitations. There are one or even two people in the institution. Two people combined with self-motivation is not enough, so it is very loose to make these available in each cluster. This is the case. All the health extension professionals are talking about TB contact screening, contact screening, and the children who are not receiving prophylaxis are being screened and monitored. Trainings are shortened or rarely occur. Health Extensions in particular are rarely one of the few, so they do not pay attention to this region like other activities, except for sending those who show symptoms. There are also barriers due of resources shortages. But it's not just the lack of income; although it is a contribution, reagents and samples, even contact screening goes all the way to the house, we have reagent shortage at the institution where we do it, we often face interruptions... In order to compensate this for doing the same, we have not served the people as much today. But there is need to add this by calling them a referrer or by filling out the referrer form as requested. But at least if they send us a list of contacts, even on a holiday, after we told them that they would come with the reagent, it would have been possible to address people when we exchanged information. It has not happened yet. So we say that we will provide services to TB patients when they come to more institutions. The same service will not be interrupted if the focal point is not available; but there are some things that pass the time. I think they have an appointment today and tomorrow; there is a certain gap in terms of working with this institution. They are used to the same service, but today, it's my appointment, I'm taking appointment on this day, so there are so many gaps from time to time. We take the following: There are gaps, we are doing our best; Here's the thing. Maybe it's because we don't get many contacts from the health department, but because there are other patient flow or people's flow that doesn't match the professional; An expert, for example, has a laboratory and it is becoming beyond his capacity to handle the normal case; There is a lack of human resources, not only from the focal point, but the laboratory technicians themselves are not that much. Even if other professionals now provide more training on TB smear or this and other TB procedures outside the laboratory, it could be done through outreach addresses. It will be difficult to address this. It is difficult to leave the facility because it is a laboratory 24 hours a day. Now a couple of health centers are isolated; I think he was tested a lot. This is a big challenge. There is a shortage of human resources in the area or in the area of human resources; what he said is right. So, I will send the health extensions to the laboratory, and the laboratory will deliver it and leave it on Sunday and Saturday night. We will try to give feedback. But we want to giving feedback to our health centers, including the responses of the people they sent who were killed, the people who did not come, and sometimes the people who said they were infected with TB at Kebele, and in the planning process, they did not send normal suspect TB; or we will give them. But this is not enough. As you said before; now, for example, the focus is not blocked, let alone giving feedback, it is very challenging to monitor the patient himself, so we don't think that the connection is that tight. The district does not see our health centers. It only supports health centers. And the health center just kept the schedule or the time to deliver the health checks, and there is a gap in the exchange of information. All this is happening because of the lack of human resources from above and below. They leave it when the health works multiply. Here too, because the same treatment and other services are available, the lack of human resources has a big contribution. In my view, the workforce environment has a factor. So that we don't hide or find them when they come here at the time they said; Just come, the ones who are supposed to work for you this day, there are problems to provide service in style because it overlaps with other customers. This is not something that can be denied. Not even contacts, whether they have symptoms, whether it's pneumonia or another viral case or something other than TB, the kind of cough that they always think about. Asthma and other things outside of TB We try to screen everyone who meets the criteria, but whether they are found or not, after being examined once or twice, if they have another disease, or if the previous pneumonia does not seem to turn into TB, what is the problem with us or our clients? Moreover, if there is a better institution than ours, we will send you there and show you, but they are not so happy when you are told whether you are satisfied or not. What do they call this? TB was not found, so when I wake up from now on, there is something like this. There are bored customers who say that they did not come to me before today, because it is a disease, and we also know the same thing. We try to work together, but they don't say much about what is right about it. Another general view from the professional institute's expert, especially from the expert's point of view, is that the expert's have no aware of TB cases contact screening, in the above introduction, the expert said something outside the focus, as I mentioned before, there was not so much attention about TB. The knowledge of the patient's situation is that knowledge, whether a professional is trained or not, at least the one who is technically in the unit, the clinic service immediately, but it does not seem to be difficult to know the TB patient or to make him serve, but if he does not take the training and does not pay attention, there are gaps here, as much as the TB focal, as well as the head of the health center. It would be good if all the professionals, the midwives, and all the professionals who work in each department, take the attention that this is a concern and share their knowledge, but the attention is that much, so the normal is right about it. Even when I ask some experts, if the TB patient doesn't have a cough or cough, everyone who is related to him will rarely lose an expert who says how important the screening is. So, as I said before, what made us not be committed is that direct OPD is always an overlapping task. Because it is not only deprivation, it can be said that more attention is deprived than the same attention; why is it busy? And if there are not so many others, TB is also a focal program, so I try to pay attention to it in some of the time I have, but we can say that there is nothing that jumps out of attention. We just didn't pay attention to it. What does that mean? If I work, it's not a living. Let alone these contacts, it would be possible to address other people in every house. If I had a free laboratory, if I only did TB, it would be possible to overcome the problem of service delivery at such a level of commitment and motivation. The work over load is the same professional and the lack of attention and the professional who pays attention also has his own workload. In general, all this overlaps and we have not solved this problem for the people. TB patients usually don't believe that the treatment will be given, but trust them, especially the contacts they live with. Anyone who is with him does not think that because he has TB, he will be vulnerable tomorrow. What's the problem with the fact that there is a chance that I will be treated if I am cured? Those who do not have the mark are still healthy; there’s nothing wrong with me and I'm taking medicine, so what's so difficult about it? How come he said in a situation where there seems to be such a recognition gap? Besides that, not to mention contact, the people are farmers; most of the people we serve are giving priority to their work and not after the most obvious signs. It is almost impossible to say that they will come here. Even if it points to a lack, we don't understand or they don't pay so much attention to their work. There is a problem with this, but this is not their own gap. Maybe even after we bring them, let's believe, will they have TB or not? We screened the symptoms together. Now, especially in the area of pulmonary TB, it can be transmitted quickly. The contact increases the risk. That's right. Today's is like this, but tomorrow doesn't mean it won't happen tomorrow, even if it's a month or two months after the person has been treated for up to two years. When people often get TB; when we see the community as a community; this is family. The work priority that I just talked to you about is nothing. This is family. But if a person is infected with TB, even we, the experts, even now, even if it is said that MDR has TB, it is just fear. Similarly, if a neighbor or a person living in the area is found to have TB, they should go to Dardarun. or failure to appear; Even if things are not as bad as this, living in a state of social isolation; And the people are now embarrassed to come to the contacts of the same family; All the contacts went for TB test and I think they are afraid that the whole family is infected. They say that it is like an institution where all the families are contaminated; it is said that they are being investigated as they go. These kinds of things are terrible. The situation exists. In addition to that, when the patients themselves come, it is always the distance, so when the two-month medicine is given, they are made to take it under direct observation by professional supervision; But since it is far away from being very mountainous; they thought that they would come down and come to us, because the patients themselves have to be treated, and they always beg us to wait two or three days. Do not come all the distance by transport. The problem in the area of transportation is that the first is the cost; always not affordable, and the second is the area where transport is not accessible, so the distance from the institution is very difficult. The position, the distance, the money, all this. In conclusion, there is our own view that makes us to go behind or falling behind in this task has become a general problem. Each of us, like me, as an individual, as a focal person, from the top, from the office to the lower community, the biggest part is ours. This can be done one day. 21 people a day screen Argo; One day, if we listen to each other, if we join the community below; It is a task that can be done in one day, but the problem with Embulele has led us to this. Let's make this tutorial and work together. But even if the same problems still exist, there are problems that have prevented us from solving them. This is what I have, maybe if there is any more questions and I know it, I can add it. But what will happen to me in my view? What will happen with the boss? What happens with the expert? What happens to the government or the supporting mother office? What will happen with Health Extension? What happens to the user community? What I said is what I saw. I said not only what I saw, but what I actually did not do. This is the information I have, so the function is the same as I have given it.

Interviewer; thank you very much for your explanation and your experience. Perhaps I would like you to add one here. The feedback on the first feedback is mostly given to you and the checkpoints. And perhaps monitoring and control to you and the health center. If you add me here; it means you got up short.

Focal 1; No, problem, I tried to explain it in a certain way the district our feedback provider does not provide feedback on health posts. They provide feedback for Health centers only or for me. And they do things like this once a quarter or so when the screening activity falls. But there is no scheduled feedback. This is on our side. Now send the suspects, wake them up on Sunday, we will tell them anything. Even if they don't give us the mission we give them; sometimes, when tasks are evaluated, it is boundly evaluated together, even though it is not so satisfactory in that sense; we give some times feedback. But it is not as much as we expected. This is the exchange of information: We have the gaps and the causes of the gaps are listed earlier. This is what I have.

Interviewer;. Thank you again, maybe one at the end; We are now looking at active case finding to control TB in the health system, so maybe if they can't come, why don't you go and get tested through health extensions? If there is a problem, let it be here?

Focal 1; ok, that's right. Even there is an indicative of presence of contacts, it is must to go and screen every one. There is only one laboratory technician. Together with the laboratory technician, we tried to reach certain areas. As I said before; Due to the lack of human resources, the health extensions cannot be examined by themselves beyond sending; or they must be trained regularly. If the patient is unable to come to the institution to work there, the health extension workers in every area and every health center should be trained to take the sputum beyond TB symptoms and send it to the doctor. Therefore, if they gather and call us, we will not do so much. As we have tried before, the routine activities in the health center does not give time the laboratory. I have not been trained in smear, so there is no such thing. Normally, the reagents do not have much enough. We may move to home, or we may do campaign work because they are not satisfactory, even if we bring them, it may shorten us; what does he say? So it had to be done. We believe this there is no question. Society is suffering; If they didn't understand, we had to go and address them. But whether it's due to the lack of expertise or our own gap, so far we have not done anything other than saying, "Let's go and find out the address of the community and send the trigger." This is the same thing.

Interviewer;. Hey, thank you very much. The information you gave me to summarize is very good. The problem is mainly to check their families for TB. The main problem is what they told us when we looked at it. There is no training provided by the government. There is a lack of resources and there is a small problem of readiness in the institutions. There is also a problem of not providing feedback to the health check. There is a problem of lack of human resources. From the professional's point of view, the work load is too much. The TB patient's family lacks knowledge due to the lack of training, and from the perspective of the patients and their families, they see their work as their work. And when it continues, the community explained to me that if the whole family goes and tries to get tested, there is something to isolate them as having TB. Maybe if you have something to add or something to say let me give it a chance? If not, let's finish

Focal 1; I don't have anything new to add. Maybe many times when doing studies like this, if problems are solved first, problems that can be solved easily, core services are behind us, maybe I just say good luck. Thank you, and in the future, when we have a question, we can do normal consulting work based on the set method. There is not this much method to solve the problem, but in this study, it is considered that research that solves the problem will be done at the level of individuals and turn to the community so that it can come to the community and give feedback. Researches must be able to help us, because they need to reach the society, sometimes the society says nothing about the consumption of paper. This is a situation we have seen too, so that the problems are not piled up.

Interviewer; Thank you very much. God bless me. We are done. Thank you

**F 2**

**Coding of speech of focal 3**

Interviewer; could you explain to me the obstacles that prevent people who are in direct contact with a pulmonary TB patient/their family from being screened for TB disease.

Focal 2: Thank you, maybe it's been 3 years since I became TB focal of this health center. In general, from the angle you told me, from the point of view of the **health system or the government**, there is a lack of monitoring and control from the health system or the government. That is to say, in general, there are no permanent and continuous support and follow-up about ways to explore the family TB health situation, at health center level. Health care workers don’t support to health extension workers. To sum it up, we have a gap in the mobilization of health extension by increasing the visit and strengthening the support. In the second step, in general lack of Trainings. In connection with training, now especially the so-called health extension experts who reach the community, health extension experts with 15 to 14 year service are On what they have been trained in the past, they do not receive the new training updates. Secondly they are working by past knowledge on the TB situation in the district and there are no updates, like this. The district focal points and the whole system under the government are a bit weak. Therefore, there is a gap in providing update and training. Therefore, from this point of view, they are recognized now when we asked them in the past, we did not train them. We should have updated now. They call it like this. And because there is a gap in training, there are gaps in between. There are other gaps in terms of resources. In general, there are shortages of reagents in our health center. When we say reagents, we are now looking for a tube for gene expert. There are interruptions, even to make contacts screening, to do it. Another thing is the readiness of institutions and not being dealt with quickly. Sometimes there are also shortages of human resources. When there is a shortage of human resources, I was not trained. Even if there are problems, due to the lack of manpower, sometimes there are situations where cases are exaggerated. From this point of view, there are things that do not come. This is what we have discussed with the health center expert, who means: No other continuous feedback is given at the district level or at the health center level. Today, we have this case, they told me today, in particular, there are so-called health extension referrals, they don't strengthen the TB referral, and the second thing is that the ones we gave to them were screened. However, what happened is that I will come to me later because of the workload. As I said earlier, there is a shortage of human resources. TB, well, it would be good if everyone and all professionals were trained in TB, because TB work used to be done very intensively in the past when there were NGOs, but now the government itself does not allow it once a year because of the lack of budget, like any other general performance. He evaluates, but there is no work to evaluate TB alone, in this sense, it is a little weak. Second, if I have a lack of expertise, there is no trained professional. Now I am the only one in this health center. In terms of looking for the contacts, it means that if there is a trained workforce, it will be available even if it provides additional support and monitoring. There are also transportation and budget shortages to go to their homes to check contacts and provide health education about TB situation. There are also health posts that take 5 to 6 hours to get around, and there are budget constraints because there is no access to transportation. From the perspective of ***the health professionals,*** those who have knowledge about the condition of the TB family will be tested. Of course, there are those who have no aware. There is not a lot of time to explain the benefits and harms of their lives and get them examined. Some of them are experts. If they are experts, I will tell them that it is good if I have it, but they will bring the same. If they are far away, I told them first. There is a lot of work load, TB isn’t doing it as a task, not everyone works on it, just like all the cases that come in at the OPD level, it is treated equally. He wants them to be screened for treatment, but it is not the same as the workload we are dealing with as a remote health center. There are problems from **TB patients and contacts** that are also seen. First, they don't have the awareness that a TB status survey will be done. These people, especially those with pulmonary TB, are required to be tested. It is found in sputum, especially because they have no awareness. And what has prevented them from getting awareness is ours. Even if we tell them, they don't expect that they will not bring family, pay attention to their daily work and catch it give priority to their work. Even if we explain them well, there is nothing to come back the other burden of their families is giving priority to their work, but they often forget to bring TB and get tested after they leave. In general, there is no have no intiation and motivation in the community that TB is a killer, even though we have gaps in everything, the community is not well understood. There are problems, as I have mentioned to me from our focal and the experts, because of the overlapping workload, the commitment and motivation is very weak. As far as I can see, this is because there is more overlapping work. In general, there is a lack of man power. In general, there are things that arise from **social, cultural and economy** point of view. When we look at them, as I said earlier, perhaps I have mentioned the distance from the family level. It's steep, it's a mountain, from this point of view, there is only one person left, many times left to be examined, other social support, since TB is said to have been found, the society is talking about social isolations and discriminations, like this, especially weddings, associations, there is something to prevent TB patients and contacts from participating in different social life. If someone coughs, the community considers as because of HIV infection, there is a habit of saying, ``this person has infected me.'' Absence of these things can be said to be obstacles to the failure of TB screening and its work well. In summary, this is what he said. If there are any other questions, I will get back to you. Thank you.

Probing; I also thank you very much. The information you gave me is good. Perhaps I would like you to expand on it. There are a couple of things. In terms of the process of giving feedback and your support for the health centers, especially in terms of active case finding, you have certainly raised the issue of supporting and going home. It is possible to send them to the area. Is there not enough laboratory or if there are any trained outside of yours, please add them to me? More on support and feedback to health post and to you? as well on manpower?

Focal 2; Thank you, ok. First of all, for you brought up the TB work at the district health office level. They consider budget as root of problem, the support and supervision they give is very low, they only support those in the general health system, not TB. It is there, but they **don't pay attention** to it. It's a matter of numbers. They don't have technical support. They just fill the checklist up and leave. Also, the lack of budget means that they will send what is there, even if they say so, but it is too big to do this. Of course, the district has about eight health centers to make it accessible and support it. We sometimes talk to them on the phone, but it is very little. They say nothing, but what is there. We explain to them that they come down to work together, but they are not helping us much yet. We only meet once in three months for feedback. Feedback means that feedback is not given to us every month, but it comes to us once in three months. There are many challenges that we are facing and many things are holding us back, but before there was a program called Heal TB. They supervise; they come with the district officers and supports TB alone. They used to do a lot of work. But after Heal TB came out, there was one called Challenge TB and he was doing a little bit of moving. Now, after all NGOs are expired over, the government is not considering it as a task. It means the work. There is no **~~review meeting~~** even once a year. Evaluation is In general, it means that at the district level, we will not review the TB level itself quarterly. There are weaknesses as an institution, and as a health center, we review the report every month. When the TB work fails, we talk to the experts about what to do. Health Extensions have the same thing. They want us to put on a different face because we were born from the community, they look down on us, they don't consider us to have ever known us, they never accept us when we tell them about TB, the community has another social life of its own, but when we stand at the church, they don't listen to us. Because there are many challenges, we have not been able to achieve that, so we are talking to the manager about how we should overcome it, and as experts, we are going to the pulmonary TB areas called Pulmonary TB, and we are going to do TB screening. In addition, we only have one laboratory, we only have one laboratory, and it is very tiring to close it and make a screen. Maybe there is no one trained in slide fixation at the health center to collect sputum and bring it back. He is the one who works on Sunday and Saturday. He doesn't have time. He doesn't have time for a permit. He said that they don't hire him. The same district has four or five labs at the district level. We don't know if there are four or five laboratories in rural areas, but some of them only have one, which is a gap in the district, so if there are at least two, let's take that boy out and fix the slide here. He could have brought it and worked with the health extension, but there is only one. There are many maternal programs; there are many OPD programs, and closing it down means something else because TB works here as well. Job loss is also contributing its share, this is the general situation.

Probing;. I thank you to the information you have given us is good. In general, you have mentioned four problems in order to prevent TB families from being screened. In terms of lack of commitment and motivation, in terms of not taking training, the workload is like working in another class apart from the TB class, and in terms of the patients and their families, there is also a problem of understanding that they also see their work. From the social and economic point of view, there is the same distance that prevents the patients from coming to the institution, and there is also a financial problem to come by transportation. Also, there is exclusion that has not been resolved yet, so let's make it short, you have touched these for me, maybe you will add it in the end or if you can add anything else?, if not, let's finish it.

Focal 2, thank you, as you may have read in the introduction, as far as many things are concerned with solving problems in the community, you should also take this, especially if there is support from the district, and if they support us, we will work with the same human resources that we have, especially the previous challenges, the things that were raised at their level. So that we can work together, you have taken the same as the recommendation.

Interviewer;. I also thank God

F3

**CODING OF THE SPEECH OF FOCAL 3**

Focal 3: We intend to divide the TB patient's family problems into four parts, first from the perspective of the patient and his family, second from the government and the health system, third from the social and economic, and fourth from the health professional. When we see each of these, there is a a problem of awareness between the patient and the family. They take it as ignorance. Now, for example, we don't go door-to-door. We don't give education to the patient. Only the patient knows that the family is infected, so they don't know. What will it do for me? They think that I can go there and not die. This means that they don't know. Third, they don't have the intiation and motivation. Thirdly, there is a shortage of professionals in our institution. If they leave once or twice, they may not be able to find them. Because the professional is only one, they go to another place because of the workload. Therefore, they giving up sometimes and this is the reason. It doesn't provide support. It doesn't provide training to the experts on what time it will be given. For health extensions, our support from the health office and the health institution is weak because we don't focus like others. Third, professional training is not provided. Now I am the only one who took it from the institution, and I took it before. Now, the expert does not know what the update is. There is no health extension, neither the expert nor support. Next, there is almost no resource. It is very difficult. The **TB class** must be separated. In this sense, there is no register. The TB itself needs a lab request. For the TB register, we can say that there is no such thing. Let's go to the reagent at home, even family testing, let alone at the institution. Some of those who come are sent by the health extension. It is very difficult for them. In this sense, there is a problem. Moreover, there is a shortage of human resources. Now I work in the TB focal children's department. I go to the OPD and injections are difficult. The current institution, the government itself, has not been able to alleviate the shortage of human resources. From this point of view, there is a problem. Again, the laboratory is only one as our cluster, and then the health extension is also single in each health post. There is a vaccine for parents. They focus on it, but not much on pulmonary TB. Because there is a lack of human power, they may have no aware about contact screening since there is no training. The government, thirdly, I will submit, the district does not call for a **review meeting** or give an evaluation. In terms of evaluation, it should have been given every three months or every quarter. From this point of view, there is a problem of Feedback Now, the district does not give feed feedback when we send zero. There is also the work load for health extension worker and professionals. It may be because the district does not do orientation. The district itself is not sending us the TB report feedback. What is it? Or they did not call and evaluate every quarter; we can say that they are died. They are focusing on children, considering that TB is a killer, it is decreasing attention now. The professional does not have the commitment and motivation. There is burden because of the busy work load. Now it is called health insurance. There is a shortage of professionals because the case flow has increased so much. There is no commitment and motivation because the support has come from the district itself is low. There is no commitment and motivation. They have not received training. From **the perspective of the family and** pulmonary TB patient is lack of awareness and we don't **~~teach health education~~**. It's difficult because we don't teach health education every day. It means that there is a problem with the health professionals. He has the workload of all the professionals. We don't work there, TB is getting down to us like that, now there is a lack of awareness, now the professional is called training, so there is no, in the past I was the only one who was trained, we still don't know what it is, there is no training, or even if they were to take training, they could have called us for an evaluation and given the product like this. No, there is nothing, from this point of view, TB is decreasing, but the people are getting infected, we have a problem with the health workers, this is what he said to the experts. There is a distance. Now our district is mountainous and rugged by nature, it is too far away and transportation is not suitable. Besides, if it is not for human power, they will not come there themselves, and this is one factor. It means that if a person is infected with pulmonary tuberculosis because it infects the whole environment, it is contagious and because it infects everything, they are isolated. There is something called isolation. This is one factor that we have not been able to solve yet. The third is the economic problem. What we have taken as fourth is the perspective of a professional from a health professional. The amount of work load of commitment and motivation is usually a person's health insurance. What is called TB? He is giving up hope because he has left the thing called TB. He is leaning towards others and there is no training. They don't know what to do to keep them calm, their weight loss, coughing at night; they don't know this, unless we keep quiet and send them what we learned in the past, they don't know there is no such thing as training.

Probing;. Thank you. The information you gave me is good. Maybe you can add it to me. There are problems so that they don't come. What is the problem that you don’t go up to home and screen?

Focal 3; Even for us, transportation is very difficult, the place has its ups and downs, some say they take motorbikes up to 400 birr, and some say that it is not worth the mountain. Go there, what I said is no, there are ups and downs and fatigue. Stay here and then work. You are focal, or reception work, there is no such thing as campaign work. Except for our immediate contact, OPD or Under Fife injection, we have never done it because there is a workload.

Probing;. Thank you again, maybe there is a lack of awareness on their part, is there health education on your side?, and is there health education on the creation of awareness?

Focal 3. No, when there is campaign work in every kebele, we said to the community high lightly, If you have cough for two weeks, cough and weight loss in the kebele, health extension write refer and you should come and get tested. Unless it's a coincidence, sometimes they get little health education.

Probing; thank you, so as a general summary... Finally, from a social and cultural point of view, is there a tendency to go to a traditional healer, not paying attention to this?

Focal 3: I didn't even know this, we didn't evaluate this, we didn't evaluate anything that said they will go here, and I didn't report it because I didn't have the recognition. Of course there is a problem, but we didn't report it.

Interviewer; Thank you. Finally, let me know if there is anything you would like to add?

Focal 3: In summary, it can be said that the government / health sector TB is almost abandoning the lack of training and orientation experts. Since we are not looking at TB in the health sector like any other, I would like to say that it is on them.

Interviewer; Thank you very much

Focal 3: no problem, thank you too

**Officers speech coding**

Officer; Obstacles There are many obstacles; As you mentioned to me, as you want to cover, there is from the government, there is from the professional, there is from the community itself, there are others, there are others; And if I try to mention some, there are many obstacles due to shortage of adequate budget **from the government**. For example, there is **no waiting room** in each health center for coughers, so there are 8 health centers in our district. And the lack of a waiting room is an obstacle. The TB room and the service room are sometimes even separated now, there were health centers that provided the TB service together with the patient examination room. Even the separated class is not enough, it is narrow and with no windows, in which the service is being provided. The other one is not being given enough training for whom is adequate training? Even for the professional, there is a chance that a professional from the health center has not been trained. Besides that, there is an opportunity to become a TB focal with no training. That is, when a professional is in permission, another untrained professional will provide services. And because the trained professional's belief that they should be screened when enough contacts arrive is not the same as the trained professionals, deficiencies are created when the untrained professional is given. And there is a lack of training. Other, health extensions are not adequately trained on how to take samples and screen others. It is the training they took while they were in school. If it lasts for a long time, there will be something left from providing adequate service. But they are not trained in TB. On job contact screening training is also not given for lab professionals. The health center in the city is the health center where they are said to have received better training from one health center. There are some one, four or five labs that have taken part. There are labs in other seven rural areas that have not been trained in AFB or TB. The rest will not be evaluated and reviewed. **Focals are not called** every quarter and professional health extensions are not called and evaluated like this. How are you doing How do you screen? How do you treat TB contact lenses? It is not evaluated. This is related to the government because of the budget. There is another lack of human power. TB focal also work in pediatric examination and other departments other than TB class, others also contains an overlapping focal due to lack of adequate human resource. He works here and there, he didn’t focus on the TB department so that contacts could come and be examined; they should be checked every three months for two years, but there is a situation of non-delivery. These are the ones that are relevant to the government as there is **a problem to pay attention** to. Duty on duty is not being paid in addition. For example, there is a ten-day salary for the laboratory. Like our district, they are required to work for 38 days, but the ten-day salary is not enough. In my view if they were worked full time, it was possible to go to home and screen contacts that could not come to institutions, work on accepting samples, giving samples. There is another barrier from the ***health care workers***, There is a lack of commitment and motivation with the professional, Lack of concentration: work as a second job; In order to associate him with the government, because there is a lack of man power, they said that it is the work of focal point of TB; Without bleeding TB's work with determination; Instead of saying I will not move to another; There is a situation of abandoning this and going to the other parts without doing what should be done enough. There is a problem that can be seen **from patients and contacts**. The patient was coming and given a tests at the time of his distress, whether it was provoked by others or by a professional, or he came and was tested on his own. After being diagnosed with TB, he didn’t thought what kind of pressure will be created from my family? There is a problem of neglecting and non comitment. He gives priority to their work and goes to his other work. He doesn’t brings his family contacts for screening, not examined those who are closely related to him. This problem from that of the patient. Another problem is the **social and cultural related**.it is very distant between contacts home and the institution. There is up and down, mountainous, it's boring; the road is mostly this problem. Another problem is that there is a situation of social isolations and discriminations that sometimes happens. Instead of saying that if TB is found, he should be careful and stay with us. Instead of saying that it might come back to us, it is sometimes seen from a situation of avoidance. On traditional then; There are people who say they know where to trust in culture; There is a tendency to go to them. the traditional medicine area It can be seen that there are problems like this, there is a lack of money transportation in economic terms, but while the transportation is very expensive, if they go by motorbike, they can now ask for a hundred birr or two hundred birr.

Probing;. Thank you very much, honorable TB officer. It is a good idea that you have raised. Perhaps I would like you to explain it to me. There are 8 health centers and 33 health centers under it. Perhaps from your side, from the point of view of monitoring and control, from the perspective of input, perhaps from the perspective of input, perhaps they have seen some focal points. Support and monitoring can be continuous or monthly, monthly or bi-monthly. On your side, we have talked about monitoring and control and resources, maybe reagents and sometimes even classes. Reagents and request form registers.

Officer, what is the monitoring of these 33 health posts and 8 health centers? If possible, it would be good if we could monitor them every month. Otherwise, we would have to monitor them every quarter. There is a situation where we are not following up on the situation. There is a remote health center that means you can't support it even if you go every two months or every month. You mentioned earlier about the supply, there is a shortage of supply, there is a shortage of reagents, the reagent is not prepared at the regional level, it is prepared like this at the regional lab, it is distributed to the hospitals, but sometimes they face supply problems and they may not deliver it to us. It is being interrupted. We still have a problem with the Felken Tube for gene xpert, registration books although repaired.

Probing;. Thank you again. Maybe it's because you will support it even if it's a piece of feedback. Maybe it's the health center for the health post. You told me that you'll support it even if it's piecemeal. How was the feedback?

Officer; I have already mentioned it before, when I raised the budget situation with the government in connection with the budget, we are still facing paper problems related to the budget, and we are still facing it. What is required is that it should be sent in hard copy every three months, but it should have been sent as soon as possible, but now there is a situation where I will send it every quarter or every 2 months by telegram, but I am preparing it in hard copy. It is impossible to say, but we will give the same by telegram, even by hard copy. Now, I have not given it to him in these six months, and I mean it to him. As I told you, I only sent them by telegram because there was a problem with the paper, so if they don't use a phone, it means there will be a problem seeing anything, and the hard copy would have been preferable. But because of this, I didn't support, I mean I didn't give feedback. When I supported, the health center focal points said that they either give to the health center or not. The problem is that some of them lack attention because of the reason I told you earlier, or because they are not being evaluated and there is a lack of expertise. I know there are experts who overcome obstacles and give feedback. There are TB focals that I get without giving anything. Along with the other problems, you will also find experts who have a lack of attention. This is the kind of support and monitoring

Interviewer;. Thank you very much. The information you gave me is very good. If it needs to be simplified, perhaps it is from the perspective of the health or your office. There is a lot of work, the priority of their work, their health, and there is a neglect of what will happen to others if I am saved. Also, from the point of view of the community, from the point of view of the economy, there is a long distance in terms of transportation. Is there any additional?

Officer, what I want to say in the end is what I want to say about what should happen next. TB is a deadly disease. Why is it spread from person to person? Even if we assume that they are vulnerable, if there is a period of 8 hours, if a pulmonary TB patient is in contact with another person for any reason and spends 8 hours that person will become a victim, and if he is not treated, after becoming a victim, he will die if he is not treated in time. First of all, the professional must be committed and give continuous health education and create awareness in the community and the problem must be solved. The prevention of TB should be reduced. If the supplies from the government that I mentioned before are adjusted, the lack of budget will be given to TB. This should be done. TB classes should be done. If the expert creates awareness, I have a message for the lack of commitment to the community.

Interviewer;, thank you very much. So, as far as possible, all these problems are solved by the government, by you, by professionals, by the community. It is the dream of all of us to see the society free from TB health and TB problems. Thank you very much for the information you sacrificed so much.

**Coding of contacts speech**

**Contact 1**

Interviewer;. Tell me about your experience, what are the barriers to screening for TB status by health professionals of people who live in the same house with a TB patient?

Contact 1; the problem is that there is too much problem from the point of view of health and the **government**. What is the problem, they don’t support us. For example, it seems that there are TB focal at health posts, they don’t help us. At least on our environment; now, even on Sunday, from Monday to Friday, the farmers go to work. They even don’t come on Sundays and say that the so-called health extension workers who circulating and give the vaccine. They don't recognize and support us on the Kebele. Care and support are not given to the farmers. From this point of view, there are some cases of family exposure to TB and pollution of the environment. And the government has not seen this problem: it is said that there is a focal person from the health workers, and from the point of view of the health centers, neither the experts nor the government have acknowledged this support at least. And there is a great problem with the government body and also to; the second and still, we still lack support and follow-up. I am surprised by the lack of resources. He is an old man, my father. He is at least 60 years old. And we have come with this old man TB patient in a very rural and faraway place. They say there is no resource, come back. How is it that we now have a 3-hour hike, there is no car; what's the matter, we climbed the mountain and fell down on our feet, and there is no resource for this old man? Even this old man is being told that he is not there when he comes, and it is time to stop taking the medicine. And the resource problem, whether it's the government or the institution, we don't have recognition. We are farmers, there is such a thing. Secondly, even if the resources are provided on some days, they are not dealt with quickly. There is a reason why they are being called "Kele Gbi". Well, at least for us, the 3-hour journey is the nothing. They sometimes tell us that it will take two hours in the morning if they start in the morning. We came at ten o'clock in the night and reached there even two hours from the institute. There is something like animal when we come in night,. But they are not there yet in time. It's three o'clock. Second, she has permission and go anywhere. There's no time to stop pushing. They say there is something, she left, she went to her family, and we say whatever it is, or come back tomorrow. When we tell them, how are we going to go back tomorrow, they say go to the manager's office? They push us there. There is a problem; there are problems on serving very quickly, so there is something like this, they say there is no key. Second, and now, for example, in our neighborhood, I have reached the seventh grade, sixth grade, seventh grade, I have learned some biology, some TB, they used to tell us the symptoms of people with TB, and sometimes when they told us that someone else had something like this, he said sputum. When they tell us that you are free when you are tested, send the laboratory and you are free. We are **saying that you are free. We are showing signs** like this. It is a sign of TB. We were given an appointment with the lab class. We were given an appointment for that day. Then there is something called a phone number. I happened to give them a phone number, and the next day, when I was sick again, I went back. I have seen by them, even more than them, my doctor says, and it is being said that you have nothing, we are being hurt, give us another drug, there is no other solution, so we are being hurt a lot. Again, secondly, from the point of view of ***the health professional,*** there is still a lack of commitment and motivation. They have learned a lot now. They have studied for seven years and become doctors. They have studied for five years and six years. We were expecting them and nothing will get better again. Farmers, we farmers will get better and they have no such commitment and motivation. Their attitude is just zero. Let us go. If they do not accept us with hope, then again they will raise our behavior, if they will ignore us, if they will not give us direction. Just like how we normally work in a normal farm, I can see that at least they know a lot about the treatment. Secondly, there is something called workload. Work pressure, I'm so busy, I'm busy, I'm going to go to another class, but this is the truth, here is the TB class, one expert, one expert is not enough. At that time, TB needs a self-sufficient expert, not just one expert. If you have something, you can get it from the government or from a body that looks after you. When a person lives at this time, he may face many problems. Of course, there are problems with you, the professionals, and with us. A person may get sick. The family may call him and go. At that time, if a person is isolated, how are the patients doing? So there is something called workload. He can go out to drink coffee and someone else can call him and leave. At that time, he must be replaced by another person. Secondly, he should not be given another job. In that class, there is something called TB class, isn't it? TB focal, what do you call it? No other workload should be given. For example, I am tending to cattle, plowing the field, am I cultivating the field or am I tending the cattle or the field will remain, or the cattle may go to another crop and they may harm us. It means that I am a farmer and cattle just like us. You will take care of us

At the very least, you, the caregiver, must have good manners. In view of this, there must be .So for workload; For TB, let's say two people, three people, four people, and they don't have another job, they don't give them another job, please tell us this for the concerned body. **When it comes to us now,** we are mostly uneducated people and we didn't know how to diagnose TB, we are no awareness about screening contacts. My families used to say that it's a new disease, we don’t know it. What are they saying that they are not willing to go. We are in a very troubled time and we don't know how to diagnose TB; it means that an expert legal body like you came and **~~did not teach us how to diagnose~~** TB and we have no awareness. Secondly, we gives priority to our work , and now we do not prioritize our health, which means that we all, and as you know that because of the overload of work, for example, now father is sick, my priority is not to save Father's life, but to work. We don't say that we can live today and work tomorrow. Our lack of education and lack of education this is the problem of our society and our lack of education. At that time, the government **~~did not create awareness~~** these health professionals, these health extensions, and these, at least these communicable diseases should be given priority. We should be safe and prepared for the purpose of the work. You didn't give us good results, you didn't give us care, advice, or products, and first of all, in my family now, in fact, as one person is able to come on foot, and he wait until he come in bed during very sick. Why do we prioritize it? We give it to work. Secondly, we don't have any such thing as motivation and commitment we are just boring. Our thinking is weak, we don't say that we are treated for this disease so that no one else's life will be affected, and we don't go to our work, and there is such a thing, Gash. Now we also see the situation from the **perspective of our neighbor and the community**; it means that you don't give priority to education, our relationship with our environment has fallen. Now TB, just like I hear it from my mother and father; Once it has a develop, it will not left; we also know that it is transmitted; There is something, so we are isolated in the area, Now for example; my father has TB; But while before this; when we were drinking coffee together; Association is called; There is something called senbete; There is something that is said to be very round ; We used to eat together; At least we are around five or six houses; The neighbor is calling us while we were eating. Since the day this disease occurred, we have not been how are you. There is no such thing before. No, let's get together and eat together on Sunday. There is no one saying hello: and this is just the local children coming and playing with their families and eating from the cooked food; even children's parents, our mothers and fathers are not educated, they are angry and locked up. It's a bad disease. It's not for greetings. We're not allowed to walk in the forest. We're not getting social support from the community and we're still not getting social support. How is it? It is very difficult for us to send people with TB to a place like this. The distance from our house to the health center or the health center is very difficult. It is difficult for humans to travel by mule or donkey. The distance is more than three hours. There is a lot of water in the mountains. Now the winter is coming. I am confused about how we will be. When we give them three to four months, they are saying no. The experts wonder how he will die. But our father, there is heavy water here, and there is very strong water at a distance from the road. There is no bridge for three months. The road is mountainous, there is something like this here, especially now, and sometimes when we import motors, there is an economic problem, so we are being asked for 500 Birr 600 Birr. We are farmers. We believe that when we come by Bajaj or by motorbike, we are only told to give 500 to 600 to the family. If you say give money to the family, give them grain. We don't even get we're in a lot support; of trouble because of the economy, that's all we have.

Probe;. Brother, thank you very much. You have shared with me the good problems you have seen. Thank you very much. Maybe some explanation is needed. Maybe if you know it's your problem, did you have the education or recognition to investigate the family?

Contact 1: No, I don’t aware.

Probe; what is the other problem of the professionals is it not being present at their workplace or is it an attitude problem?

Contact 1; there is also a problem with not being present on time, especially not being present on time, we just didn't realize how far away from the road they were, and secondly, they’re not being present on time.

Probe;, thank you again, very likely the information you gave me is good. In summary, there is something you have explained. We are not supported and monitored by the government or health. There is a lack of resources. I think they are busy because there is only one person there. You said that we are having trouble when they leave. Another thing is that you do not know that the family should be examined. You have not developed an understanding. Another thing is to give priority to work. Another economic problem is that there is a steep river that takes up to three hours to walk. Maybe if there's anything you'd add at the end?

Contact 1; thank you very much. You come and ask like this; we thank you for asking us for advice. The same thing has been said in other areas, and you are also coming to them. You should present your message to the body that is looking at it. It would be good if you would at least give us an education. Thank you, Gashe.

Interviewer; I also thank you. As I said earlier, the purpose of the research is to give suggestions to solve this problem without any body at every level.

Contact 1: On top of that, I just said that I have studied up to the seventh grade; but some other people and some of our families did not accept us when we told them. He didn't say anything; he just said that you have arrived today. Diet and about the purity of disease; they don't listen to us. Even though we don't know more than you, we don't have a better knowledge than them because we studied a certain amount until the seventh grade; we are a little better than them and there is just such a thing as garden cabbage. Even if you don't know, we believe him, he said quietly and they don't accept us; They don't listen to me now that I'm Fazer; Don't talk about this and don't talk about that, he won't listen to me. Similarly, they are small and do not listen to us; and they will listen to you coming at least every Sunday, especially health professionals. A public servant of a government body; they respect you, they know you, and if you advise us, they will listen to you. They did not listen to us; it would be good if you also come and give us training every two months. Our families and our surroundings are sick and we hear you; they will receive you; they are rejecting us

Interviewer; Thank you again

**Coding of speech of contact 2**

Contact 2; most of the time when I come with my brother who is TB patient, not even they screen us, the health professionals are **absent from work place**. So I think there is low monitoring and supervision. They came after called from other class.perhapes they also say there is shortage of supply, interruption of electricity. The other when they suspect and want to investigate my brother, after he come to health institution, by saying there is no working chemical they appoint repeatedly and there is a problem of resource and supply. The other probably the available health center doesn’t serve this much quickly for clients, there is delay’; although we come in the morning we don’t go back till 9up to 10 o’clock. The other I think there shortage of man power. Because a single man works in many classes, for example the man that gives drug for my brother works in other examination room and come after I searched him. The other probably on this related my brother **had symptoms but TB was not** detected with repeated sputum investigation of laboratory, they said he is free of TB and TB was detected after he went hospital when the illness increases with I don’t know the problem repeatedly three or four times he was examined but they said free . The other perhaps from the health professionals they **don’t pay attention** for us when we go the health institution and they **~~didn’t create awareness for u~~**s. As we live with the pulmonary TB patient health professionals don’t teach us what to do and what not to do, only drugs received and go back nothing done or no advice was not given for me. As I said before there is work load, the one who gives anti TB drugs to me also works the other examination room, after waiting for a long time I call him and receive drug to my brother. Also we don’t have awareness about screening of contacts, we the families are happy and wished to treat my brother after he diagnosed TB, but we are not aware of screening of us. Out of this we are busy, work loaded, we give priority to our work, when we come every day to this with narrowing the working time we consider our work not for screening. In relation to work load we don’t pay attention for screening we don’t think there is TB inside us, as well we are not committed and initiated for examination. Then in our community when there is a TB patient especially pulmonary TB, there is isolation and discrimination in the community, he doesn’t get other social service, and this are some of the reasons that barriers us from screening Tb status.

Probing; what is expected from the government related to the barriers you explained?

Contact 2; as it is known TB is a deadly disease, many brothers many people died before drug is discovered, it is my pleasure is the government supervise and control it. Firstly, permanent focal should be assigned who works on TB class only, and he should create awareness to the community about the need of screening quickly. The other we are boring of calling and searching the workers, they are not avail on their class sometimes we find them in cafe, we are waiting for a long time from morning to 7 o’clock mid-day. And the government should control them and assign permanent person for TB class to serve the community.

Probing; how is his intimation and commitment to serve you after he comes to working room?

Contact 2፤not this much committed he only give us drugs after seeing the register and asking the patients name, without come to saying tomorrow he go back without said nothing.

Probing; what about your commitment initiation and interest for screening? Do you pay attention?

Contact 2; we don’t have awareness, we only care for TB patient if there is in our environment, we don’t know whether we are infected or not. If they educate us we are voluntary and committed to be screened and taking care. After this time if the problems I raised before, like shortage of resource, solved I my self is ready to be screened today.

Interviewer; summary and if there is an additional at the end?

Contact 2; the remaining thing that I want to add is, we are in urban, most my family are in countryside my uncles and aunts, there in is the community generally TB patients are discriminated and don’t give care the patient as they thought it is transmitable,they don’t socially support to continuously take drugs. There for health workers should go up to home and create awareness for the community to care the patient and screen the contacts of the index cases.

Interviewer; thank very much!!

**Coding of the speech of contact 3**

Contact 3: Well, the barriers as my consideration, the first one is distant. We are in the countryside, it is very distant between our home and the health institutions to go and get screening. It is far from the house to the institution. In addition, perhaps, a person with TB is stigmatized in the environment, families afraid to go. It is considered to be a disease that cannot be cured like any other disease. I think it is one of the reasons. Apart from that, it also requires transportation costs to go there now. Farmers work and feed for today but they don't think that they can take care of themselves tomorrow; from the same point of view, there is a problem with the economy for transportation. This taken as a reason. In addition to this, perhaps, as I told you earlier, a person with TB is said to have cancer. I think that a person with cancer is very contagious just by looking at it. They are the things that prevent us from examining them in our own way. OK, apart from that, now regarding **TB patients and their families**; low status of awareness from our point of view about TB health. Now we hardly have the knowledge to go and be tested for being contagious. It means that we don't have much awareness. Apart from that, according to what I have already said, work over load of family; Prioritize work; motivation; mainly we have no motivation. We have no ommitment. And this is a problem from our point of view. Apart from that, when we go from the **point of view of the government**, now; Lack of monitoring and supervision. Failure to provide regular and consistent supportive monitoring of family TB status to health centers. They say that there is a lack of resources. They say that there is no electricity; they say that they do not have any chemicals. Absence of **separated TB Class,** yes, so there is no monitoring and control from the government. There is a condition of being closed when we go the institution. They tell us that there is a lack of resources. It means that there is also lack of commitment. They say we are busy. So, this is the problem from the side of the government. In addition, there is a **symptom of TB but the lab is free**. It means that they don't tell me that I have TB, but I gave them sputum and they said you are free. I have been tested in the laboratory and I can't know my results. We hear some things from the community. They said I was examined and told that you don't have TB. It means that this is a problem from the side of the government. Apart from that, health professionals are not committed to examining the family of a TB patient. Health professionals have no commitment to screen the family of a TB patient; they have no commitment to investigate. They have a lot of work. They say we have a lot of work to do. Since there is a lack of man power, most of them prioritize other work and do not pay attention to this matter. And this is it.

Probing; do they come to your home? What they do by coming to you?

Contact 3: Health professionals never come and doing nothing, may be it is far from here to institution, they do nothing.

Probing: do they serve quickly? And are they available on time on their work when you go with your brother?

Contact 3; as I said before, sometimes they are difficult. It is said that they have gone Gott. They are living but not treating. It means some kind of quarrel, there are problems.

Probing;. Thank you, and on your part, maybe you should go and investigate so that there is a lack of recognition and you have never been given awareness?

Contact 3; May be because I'm a little educated, I know. But I have nothing to say that awareness has been given from the family area. Also commonly referred to as a TB patient; He has another serious illness; there is a thought that he should be excluded from social life. Attitude: Because there are problems. They were not given the understanding; In terms of society, it means; And these are these problems; And the health workers do not give understanding there. We will not go and investigate. It's a list of problems.

Interviewer;. Thank you very much, summary and any additional?

Contact 3; Thank you; So it should be; Now, as you said before; Because TB disease is contagious; It means that people who are in close contact with the patient should go and get tested. I taught him and my family as much as I could. I will try to educate the local people as well so thank you.

Interviewer; Thank you very much too.
