# Supplementary material for: Exploring barriers of household contact screening of index case contacts of pulmonary tuberculosis cases in Sekela district, Amhara region, Ethiopia: 2023; descriptive qualitative study
Source: PLoS One. 2025 Dec 30;20(12):e0339992. doi: 10.1371/journal.pone.0339992 (PMC12753078; doi:10.1371/journal.pone.0339992)
Supplement: S1 File — (ZIP) [file pone.0339992.s001.zip › transcribed and translated.docx]

**1**

ጠያቂ፤እድሜ ስንት ነው

ኤክስቴንሽን 1፤እድሜየ 33 ነው

ጠያቂ ፤ በጤና ኬላችሁ በዚህ ዓመት የሳንባ ቲቢ ታካሚዎች አሉችሁ

ኤክስቴንሽን 1፤ ሁለት ታካሚዎች አሉን መድሃኒትም እየዎሰዱ ነው

ጤያቂ ፤ ስንት ወር ያህል ወስደዋል

ኤክስቴንሽን 1፤ ለአራት ወር ወስደዋል

ጠያቂ፤ ከእነዚህ ቲቢ ታካሚዎች ጋር ያሉ ቤተሰቦችን አውቃችኋቸዋል

ኤክሰቴንሽን 1፤ አዎ እናውቃቸዋለን

ጠያቂ፤ስንት ናቸው

ኤክስቴንሽን 1፤ ስምንት ናቸው፡፡ የአንደኛው ታካሚያችን 5 ሲሆኑ የሁለተኛው 3 በአጠቃላይ 8 ናቸው

ጠያቂ፤ ከእነዚህ ግንኙነት ካላቸው ቤተሰቦች መካከል እድሜያቸው ከ15 ዓመት በታች ልጆች አሉ

ኤክስቴንሽን 1፤ የሉንም ሁሉም ከ15 ዓመት በላይ ናቸው

ጤያቂ ፤ ለእነዚህ 8 ግኑኙነት(ኮንታክት) ያላቸው ቤተሰቦች ምን የተደረገላቸው ነገር አለ

ኤክስቴንሽን 1፤ ቤተሰቦቻቸው ጋራ አንደኛ ከቤተሰቦቻቸው ጋራ ምንም የተደረገላቸው ነገር የልም፡፡ እነዚህ ታካሚዎችን ብቻ ያው መድሃኒት እንዲወስዱ ክትትል ከማረግ በስተቀር; ከቤተሰቦቻቸው ጋራ ምንም አይነት ምረመራ አልተደረገላቸውም፡፡ምክንያም አንደኛ ከወረዳም ምንም አይነት ድጋፍ ስለማይደረግ፡፡ከዚህ በፊት ምርመራ ማድረግ እንዳለባቸው; የወረዳም ሆነ የጤና ጣቢያ ባለሙያወች ድጋፍ በሚመጡበት ሰአት ሌላውን ;በአመት አንድ ጊዜ ሁለት ጊዜ ቢመጡም ሌላውን ስራ ነው እንጅ ትኩረት እሚሰጡ፤ እንደዚህ አይነት የቲቢ በሽተኞች ጋር ያለውን ችግር ምርመራ ማድረግ እንዳለባቸው እውቅናውም ሰተውን አያውቁም፡፡ እኛም ከዚህ በፊት ስልጠናም ስላልወሰድነ ቤተሰቦቻቸው እንዲመረመሩም አላደረግንም፡፡ከዛ ውጭ የግብአት እጥረትም አለ ነው እሚባል፡፡ አንዳንዴ አሁን እኛ ዝም ብለን በተማርነው ቤተሰብ ያው በፊት ስንማር ቤተሰቦቻቸው አንድ ሰው የሳምባ ቲቢ በሽተኛ ከሆነ ምርመራ ማድረግ አለበት ቤተሰቡ ነው እሚባል፡፡ስለዚህ እንደዚህ አንዳንድ ጊዜ ቤተሰቦቻቸው ወስዳችሁ አስመርምሩ በምንልበት ሰአት ሌሎች አሁን እነዚህ ሰወች በሚመረመሩበት ሰአት የግብአት እጥረት አለ ነው እሚባል፡፡የባለሙያ እጥረት አለ፡፡የግብአት እጥረት አለ ፡ስለሚባል ሪፈር ተብለው በሚሄዱበት ሰአት መጉላላትም ስላለ ስለሚማርሩ እኛም ሪፈር አንላቸውም ብዙም፡፡የሪኤጀንት እጥረት አለ ነው እሚባለው እና እሱም ስላለ ያን ያህል ሪፈር አንላቸውም እኛ፡፡ሌላ ወደ ጤና ጣያ ሲሄዱ ሪፈር ሲባሉ የለባችሁም፤ምክንያቱም ምልክቱ አለ ግን የለባችሁም ስለሚባሉ ለመሄድም ፈቃደኛ አይደሉም፡፡እነዚህ ሰወች ሁለቱ ሰወችም የተገኘባቸው ቡሬ ሆስፒታል ሂደው ነው የተገኘባቸው፡፡ እነሱ ያው መድሃኒቱን ይዘው መጡ ወደ ጤና ጣቢያ ተላኩ ከጤና ጣቢያ ወደ ጤና ኬላ መድሃኒቱ መጣ የሁለቱን ወር ፊትለፊት ሰጠናቸው ከጤና ኬላ አሁን ከሶስት ወር ጀምረን ያለውን በየ ወሩ እየወሰዱ መድሃኒቱን እየወሰዱ ቤተሰቦቻቸው ግን ምንም አይነት ምርመራ አልተደረገም፡፡ የሰው ሃይል ዕጥረት ስላለ ;ተጠርጣሪ ሰብስበን በምንጠራበት ሰአት ፤እነሱ የሰው ሃይል የለን ላብራቶሪወች ትንሽ ናቸው; ወደሱ አንልክም ወደዚህ ይመጡ; ነው እሚሉት፡፡ እስካሁን ምንም አይነት ምርመራ አልተደረገም፡፡ሌላ የበጀትና የትራንስፖርት እጥረት አለ ነው እሚሉ እነሱ ::አሁን አንደኛ መተው ምርመራ ለማድረግም ጤና ጣቢያውና ጤና ኬላው ያለበት እርቀት ሩቅ ስለሆነ የበጀት ዕጥረት የትራንስፖርት አገልግሎትም ስለለ መተው ምርመራ ለማድረግም በጣም ስለሚቸገሩ ህብረተሰቡም ከአካባቢው መጥቶ አሁን እነዚህ ሰወች ስምንቱ ቤተሰቦች መተው ከጤና ኬላው ላይ ምርመራ ለማድረግ እነዛ ጤና ጣቢያ ሂደው ምርመራ ለማድረግ የትራንስፖርት ችግር ስላለ በጣም ይቸገራሉ ምርመራ ለማድረግም በጣም ተቸግረን ነው ያለን፡፡የበጀትና የትራንስፖርት ችግር ስላለ ማለት ነው፡፡እና ለሌላው ስራ ነው እንጅ ለቲቢ ፕሮግራም ብዙም ትኩረት አይሰጡትም፡፡ስለዚህ እንደዛ ስለሆነ እኛም እነሱ ትኩረት ስለማያደርጉ ድጋፍ ስለማያደርጉ እኛም ትኩረት አላደረግንበትም፡፡ያው ለወደፊት ከነሱ ጋር ሁነን ትኩረት አድርገንንዛቤ መፍጠር ነው፡፡ሌላው ከታካሚወችና ከቤተሰቦቻቸው አንጻር ያለው ነገር ፤አንደኛ የግንዛቤ ችግር አለ፡፡ግንዛቤ እጥረት አለ፡፡ እኛም ግንዛቤም አልፈጠርንላቸውም ግንዛቤ ቢሰጣቸውም ወደኋላ የማለት ችግር፤ለስራቸው ነው ቅድሚያ እሚሰጡት እንጅ ፤ታካሚወች አሁን ከነሱ ያለው የቲቢ በሽታ ወደቤተሰቦቻቸው ይተላለፋል ብለው አያስቡም፡፡አይተላለፉም ብለው ነው እሚያስቡ፡፡ስለዚህ ግንዛቤ ችግር አለባቸው ከራሳቸው ላይ ለራሳቸው ብቻ እሚሆን ነው እሚመስላቸው፡፡ሌላ ስራ ስራቸውን ነው እንጅ ኢሚያዩ አሁን እነሱ መድሃኒት ለመውሰድ እራሱ ቅድሚ ስራቸውን ነው ዕሚያዩ ምክንያቱም አላስተኛቸውም ፡፡ስለማያስተኛቸው በሽታው ስራቸውን ስለሚሰሩ ከበሽታው የልቅ ለስራቸው ነው ቅድሚያ እሚሰጡት ፡፡እና ስራቸውን ስራቸውን ስለሚያዩ ወደዚህ መተው ቁርጠኝነትና ተነሳሽነት ስለለላቸው ለቲቢ ብዙም እንትን የላቸው፡፡ትኩረት የላቸውም ፡፡ሌላ ሰው ማለት አካባቢው አሁን ሰወቹ ያሉበት አንደኛው በጣም እሩቅ ቦታ ነው ያለ፡፡በተለይ ወደ ሜጫ ድምበር ነው ያለ አንዱ ታካሚ እና እነዚያ ሰወች አሁን ቤተሰቦቻቸውን ይዘው መተው ቲቢ ምርመራ ለማድረግ በጣም ይቸገራሉ፡፡ አንደኛ ትራንስፖርት ሞተር ነው:: ብዙ ጊዜ ያሉ ሞተሮች ደግሞ በጣም ውድ ናቸው፡፡ስለዚህ አሁን አርሶ አደር ብር ከሚያወጣ ይልቅ; ሌላ ነገር እህል አምታ ቢባል ይቀለዋል፡፡እና ያንን ሞተር ከፍሎ መጥቶ ምርመራ ለማድረግ አንድ ሰው ቢሆን ይቻል ነበር፡፡ያን ሁሉ ቤተሰብ ሞተር ከፍሎ መጥቶ ምርመራ ለማድረግ በጣም ስለሚቸገሩ ሩቅ ነው፡ ውጣ ውረድ አለው፤ ተራራ አለው፡፡እያሉ ነው ምክንያት እሚፈጥሩ፡፡እና ምርመራ ለማድረግ ርቀት ስላለው ፍላጎትም የላቸውም ፡፡እኛም የው ዝም ብለን በተማርነው ነው እንጅ ግንዛቤ የፈጠርንላቸው እንጅ ያን ያህል እንትን አላሉም ፡፡ስለዚህ መጥተው ምርመራ ለማድረግ ትኩረት የላቸውም ፡፡ሌላው ጎረቤቶቻቸው ያገሏቸዋል:: ብለው ስለሚስቡ በሽታውን ማጋለጥ አይፈልጉም፡፡ ምክንቱም ያ ሁሉ ቤተሰብ ቢመረመር ሌላ ነገር የሆነ; በዘር የጎደሉ; ብለው ያስባሉና ህብረተሰቡ፡፡ ስለዚህ እነሱ አሁን በነሱ ብቻ እንዲቀርላቸው; ምስጢሩ እንዲጠበቅላቸው; ነው እንጅ እሚፈልጉ ቤተሰብ ሁሉ ቢመረመር ህብረተሰቡ ያገለናል; ብለው ነው እሚያስቡ፡፡ምክንያቱም የቲቢ በሽተኛ ናቸው:: ቤተሰቦቻቸው ህረተሰቡ ያስተላልፉብናል; ብለው ስለሚያስቡ ያገሉናል ይላሉና; እና ምስጢር እንዲሰማባቸው አይፈልጉም፡፡ለዛም ነው ምርመራ ማድረግ ፍለጎት የለላቸው፡፡ሌላ አንዳንዶቹ ጤና ጣቢያ ለመሄድ ችገር ስላለባቸው ው እንግዲህ ከዚህ በፊት ምርመራ ለማድረግ በጣ ተቸግረዋል፡፡ትራንስፖርቱም እንግዲህ በጣም ችግር ስለሆነ እንዲስተካከል እና እየጠየቅን ነው መጥተው እዚህ ስክሪን እንዲያደርጓቸውም እየጤቅን ነው፡፡የባለሙ እጥረት አለ እየተባለ ስለሆነ ችግር አለ፡፡ እሱን ወደ ፊት እንጠይቃለን

ጠያቂ፤የትራንስፖርትና እርቀቱ ችግር እንደሆነ ገልፃችሁልኛል ምናልባት እናንተ ሰዎቹ ቤት ድረስ በመሄድ ለመመርመር ያደረጋችሁት ጥረት አለ?

ኤክስቴንሽን 1 ፤በእኛ ያደረግነው ጥረት የለም፡፡ቤተሰቦቻቸውን ሂደን ለመመርመር ያደረግነው ጥረት የለም፡፡ምክንያቱም ከኛ ጤና ኬላ አንስቶ ሰወች ቤት ለመድረስ ሩቅ ስለሆነ ትራንስፖርት የለውም፡፡ ሞተር ነው ያለ ፡፡ሞተርም አስቸጋሪ ስለሆነ ዋጋውም በጣም ውድ ነው፡፡ ከዛ ውጭ ደግሞ መንገዱ ምቹ አይደለም ፡፡ስለዚህ እኛ ያደረግነው ጥረት የለም ምንም ኤነት ምርመራ አላደረግንላቸውም፡፡

ጠያቂ፤የሰው ሀይል እና የግብዓት እጥረት ጤና ጣቢያውን የጠየቃችሁት ነገር ይኖር ይሆን?

ኤክስቴንሽን 1 ፤ጤና ጣቢያ የሰው ሃይልና ግብአት እጥረት ሁሌም እኛ ስንገናኝ ስንገናኛ ከጤና ጣቢያው ጋራ ስብሰባ ምናምን አለን ስንገናኝ ጥያቄውን እናነሳለን ፡፡ምክንቱም ህብረተሰቡ አቤቱታ ስለሚያነሳ እዛ ሂደን ተልከን ግብአት የለም፤ ሰው የለም ወረፋ ጠብቁ ነው እምንባል ስለሚሉ ሁሌም እናነሳዋለን ፡፡እነሱም እናስተካክላለን ነው እሚሉ፡፡ሁሌም ቢሆን ባለሙም ከዚህ በኋላ እናስተካክላለን ፤ግብአትም ይመጣል ፡፡ነው እሚሉ ሁሌም ጥያቄውን እናነሳዋለን ፡፡

ጠያቂ፤ግንዛቤ ለመስጠት እነርሱ ላይማሩ ወይም ላያነቡ ስለሚችሉ እርቀቱ ቢኖርም በእናንተ በኩል ምናልባት የጤና ኤክስቴንሽ የመጀመሪያ ስራ በቤተክርስቲያን በመሄድ ወይም ከቦታው ድረስ በአቅራቢያ በመሄድ የጤና ትምህርት የሰጣችሁት ነገር ካለ ቢገልፁልኝ?

ኤክስቴንሽን 1፤አሁን እኛ በእርግጥ የጤና ኤክስቴንሽን ስራ መጀመሪያ የጤና ትምህርት መስጠት ነው፡፡ ግንዛቤ ፈጠራ ነው፡፡እና ግንዛቤ ፈጠራውን እንሰጣለን ግን ግንዛቤ ፈጠራ ላይ በዋናነት ወረዳውም ጤና ጣቢያውም ትኩረት ስላልሰጠው ዘይህን የቲቢ በሽተኞች ጋር ያለቸውን ግንኙነት ቤተሰቦቻቸው ምርመራ እንዲያደርጉወረዳውም ጤና ጣቢያውም ትኩረት ስላልሰጠው እኛም ትኩረት አልሰጠነውም ነው እንጅ እንነግራቸዋለን፡፡ ምክንያቱም የሳምባ ቲቢ ከሆነ ከአንድ ሰው ወደ ሌላ ሰው በትንፋሽ እንደሚተላለፍ እንነግራቸዋለን፡፡ ሁሌም ቢሆን፡፡እና ምርመራ ማደረግ እንዳለባቸው ያን ያል ትኩረት አልሰጠንም ፡፡ያው ግንዛቤውን እንፈጥራለን ፡፡

ጠያቂ፤ ሌላ ሚጨምሩት ሀሳብ ወይም ከጥያቄው ግልፅ ያልሆኑ ሚጠይቁኝ ነገር ካለ ያንሱ

ኤክስቴንሽን 1፤ጥያቄው የተጠየቀው ትያቄ ግልጽ ነው፡፡ግን አሁን እኔ ለእናንተ የምሰጠው ነገር ምክንያቱም የናንተ ዩኒቨርሲቲው(ፕሮጀክቱ) ይህን መጠይቅ ሲያዘጋጅ ለወደፊት ጥሩ ነገር ይኖራል ተብሎ ታስቦ ይመስለኛል፡፡ ስለዚህ አሁን ይህንን እንትን ተሰብስብ ምክንያቱም ወደ ጥሩ ነገር ፤እዚህ ላይ ብቻ እንዳይቀር ነገ ላይ ቲቢ በሽተኞች የቲቢ በሽታ አሁን ከሰው ወደ ሰው ስለሚተላለፍ ይህ ነገር ተሰርቶበት ለውጤት ወደ ታችም ወርዶ ጥሩ ነገር እንዲሰራበት ነው እምምለው፡፡ዝም ብሎ እንዳይቀር ማለት ነው፡፡

ጠያቂ፤እስካሁን ያለው ጠቅለል ሲል ከወረዳው አንጻር ገለጹጽ አለ ድጋፍ እንደመይደረግ ድጋፍ ቢደረግም ለቲቢ ትኩረት እንደማይደረግ ነው የገለፁልኝ፡፡ስልጠና አለመውሰድ ፤ የግብዓት እጥረት እንዲሁም የሰው ሀይል እጥረት እንዳለ ለምሳሌ፤በጤና ጣቢያ የላብራቶሪ የሪጀንት እንዲሁም የአገልግሎት ችግርም አለ ብለውኛል ቶሎ አለመስተናገድ ፡፡

ከታካሚዎች አንፃር ፤የግንዛቤ ችግር እንዳለ ስራቸውን ማስቀደም ለበሽታው ትኩረት አለመስጠት..

ከማህበረሰቡ ወይም አካባቢው አኳያ ፤ህብረተሰቡ እስካሁን ድረስ አመለካከቱ ላይ ችግር እንዳለ በሽተኞችን የማግለል ንገር እንደሚታይ እንዲሁም ታካሚዎች ከእነ ቤተሰቦቻቸው ከርቀቱ ጋር ተያይዞ የትራንስፖርት ችግር እንደሚጋጥማቸው ገልጠውልኛል ፡፡አሁንም በመጨረሻ የሚሉት ሀሳብ ካለ መጨመር ይችላሉ

ክስቴንሽን 1፤ያን ያህል ሀሳብ የለኘኝም እናንተ ለቲቢ ፕሮግራም ትኩረት ሰጥታችሁ ስለመጣችሁ እኛም ወደፊት ጥሩ ነገር እንሰራለን ፤እናመሰግናለን፡፡

Questioner: How old are you?

Extension 1: Age 33

Questioner. Do you have pulmonary TB patients under your health care?

Extension 1; We have two patients who are taking medicine healthy

Questi ; How many months did it take?

Extension 1; they took it for four months Questioner. Do you know families with these TB patients?

Extension 1; Yes, we know them

Questioner: How many are there?

Extension 1; There are eight. The number of our first patient is 5 and the second one is 3, for a total of 8

Questioner. Are there children under the age of 15 in any of these related families?

Extension 1; All of them are over 15 years old healthy

Interviewer: What has been done to these 8 contact families?

Extension 1; Together with their families; First of all, nothing was done to them with their families. Except for monitoring these patients to take only the medication; they have not been examined together with their families because, first there is no support from the district. When the district and health center experts come to support the other, even if they come once or twice a year, they pay attention for another job. They never acknowledged us the need to diagnose the problem with TB patients. We have not received any training before, so we did not have their families examined. Besides, it is said that there is a lack of resources. Sometimes now we just learned; Family When we learned before, it is said that if a person is a patient of pulmonary TB, it is the family that should be tested. Therefore, sometimes; it is said that there is a lack of resources at the time when these people are sent to be examined. There is a lack of professionals. There is a lack of resources. At the time when contacts are going to be referred to health institution; they complain because there is abuse, so we don't refer them. It is said that there is a shortage of reagents, and we don't refer them that much because there is a shortage of reagents. When they go to the health center, they don't need to be referred because they say we have the symptoms but they say, you don’t have tb, so they don't want to go. These two people; People who were found went to Bure Hospital where they were found. They came with the anti TB medicine and were sent to the health center. From the health center to the health post, the medicine came. We gave them two months ago. From the health post they have been taking the medicine every month for three months now, but their families have not been screened. Because there is a shortage of manpower, when we collect a suspect and call the health center, they said our laboratories are small; we will not send to him, come hither; they say it is. No investigation has been conducted yet. They say that there is another lack of budget and transport. Now, first of all, the distance between the health center and their home is too far to do the screening. Because of the lack of budget for transport, it is very difficult to do the examination because of the lack of transport. It is very difficult for them to go and do the investigation because there is a problem with transportation. We have a lot of difficulty in doing the investigation. It means that there is a problem with the budget and transportation. And it is for other work, but they do not pay much attention to the TB program. The same thing is to be with them in the future and create awareness. The other thing from the perspective of the patients and their families is that there is a problem of awareness. There is a lack of awareness. Actually we did not make them aware of it, even if they were made aware of it; the problem is that they are non comited to do it. They give priority to their work. The patients do not think that the TB disease from them will be transmitted to their families. Even, now they are doing their own work; priorily taking medicine, because it doesn't put them to sleep. And leaving them here because they see their work; Because of their non comitment and motivation, they don't care much about TB. And those people now are distant; they find it very difficult to bring their families and get tested for TB. The first transport motor cycle. Most of the time, the motors are very expensive, so instead of the farmer spending money; It would be easier if something else was called grain collection. And it would be possible if one contact paid for that engine to come and be screening. It is far away because it is very difficult for the whole family to pay for the motor to come and do the investigated. It has a mountain. Aand they create reason. And because there is a distance to investigate, they don't want to. So they don't care to come and investigate. Other, neighbors ostracize them; they don't want to expose the disease because they think that. The reason is that if the whole family is examined, something else will happen; Inferior; they think that the society. So they are now left alone; to keep the secret; But if every family who wants to be investigated, the society will isolate us; they think because they are TB patients. Their families will transmit it on to us; because they say that they separate us because they think that; and they want to be kept secret. That's why they don't want to investigate. Some of them have trouble going to the health center, so they had a lot of trouble getting tested before. The transportation is a big problem, when we are asking them to come and do the screening here. We will ask him further

​Probe: You have explained to me that it is a problem of transportation and distance, maybe you have made an effort to go to the people's house and investigate?

Extension 1: We have not made any effort to go and examine their families. Because it is far from our health post to reach people's homes, there is no transportation. There is only motor for transportation. The motor is difficult and the price is very expensive. Apart from that, the road is not convenient. Therefore, we did not make any effort; we did not do any investigation for them.

Probe; There is Lack of manpower and resources. Do you have anything to ask the health center?

Extension 1; There is Lack of human resources and resources at the health center; Whenever we meet, when we meet; When we have a meeting with the health center, we have raise the question because the community will raise a complaint; There is no input when we go there. We always raise the question because there is no one and they say wait in line. They say we will fix it. We will always fix it later, and the input will come.

Questioner: In order to provide awareness, they may not be educated or read, so even if there is a distance, could you please explain to me if there is anything that you have given health education on your part, maybe the first work of health extension is going to the church or going to the place nearby?

Extension 1: Now we are actually doing health extension work first to give health education. Awareness is innovation, and awareness is innovation. But mainly because the district and the health center did not pay attention to the creation of awareness, we told them that we did not pay attention to them because the health center did not pay attention to them. Because we tell them that if it is pulmonary TB, it is transmitted from one person to another through breathing. Always. And we didn't pay attention to the fact that they should be investigated. We create the same impression.

Probe; if you have another idea to add or something that is not clear from the question, raise it

Extension 1: The context in which the question was asked is clear. But what I am giving you now is because I think that your university (the project) will prepare this question for the future. So, collect this worm now because it will lead to something good, so that it doesn't just stay here, tomorrow TB patients will be transmitted from person to person.

Interviewer: Summarizing what has happened so far, please explain from the perspective of the district that support will be provided, but they have told me that no attention will be paid to TB. They told me that there is a lack of resources and a lack of human resources, for example, there is a problem with laboratory reagents and service at the health center. From the point of view of patients, there is a cognitive problem, putting their work first and not paying attention to the disease. In terms of the community or the area, they have revealed to me that there is a problem with the society's attitude so far, that they isolate patients and that patients face transportation problems due to the distance with their families.

EX 1; I don't have that much idea. Thank you for paying attention to the TB program. We will also do good things in the future.

**2**

**ኤክስ 2**

ጠያቂ/የጥናቱ ባለቤት፡ እንደምን ዋሉ?

ኤክስ 2፤ እግዚአብሔር ይመስገን

ጠያቂ/የጥናቱ ባለቤት፤ ስሜ ምህረት ገረመው ይባላል፤ በደብረ ማርቆስ ዩኒቨርስቲ የህክምና ና ጤና ሳይንስ ኮሌጅ፣ የወረርሽኝ በሽታ ማጥናት ት/ት ክፍል የድህረ ምረቃ ተማሪ ስሆን፤ በዚህ የሳንባ ነቀርሳ በሽታ ያለባቸው ሰወችን የቅርብ ግንኙነት ቤተሰብ ቲቢ ለመመርመር እንቀፋቶችን ማሰስ የሚለውን ጥናት እያደረኩ ስሆን የመጣሁት ለሁለተኛ ድግሪ መመረቂያ በምሰራው ጥናትዊ ፅሁፍ መረጃ ለመሰብሰብ ነዉ። ይህን መረጃ ለመሰብሰብ ከደብረ ማርቆስ ዩኒቨርሲቲ፣ የጥናትና ምርምር ስነ ምግባር ኮሚቴ እንዲሁም ከ ሰከላ ወረዳ ጤና ጥበቃ ፅ/ቤት ፈቃድ አግኝቻለው። ይህንንም ጥናት ለማሳካት የእርስዎ ቅንነት የተሞላበት ተሳትፎ ወሳኝነት አለው፡፡በዚህ ጥናት ላይ መሳተፍ፣ በፍቃደኝነት ላይ የተመሰረተ ሲሆን፤ ስጠይቅዎት በመሃል ጥያቄ መጠየቅ፤ጥያቄ መዝለል፤ብሎም ማስቆም ይችላሉ፡፡ በቃለ መጠይቁ ወቅት የድምፅ መቅረጫ መሳሪያ የምጠቀም ስሆን የሚወስደው ጊዜ በግምት ከ30 ደቂቃ እስከ 45 ደቂቃ ይሆናል። በጥናቱ ላለመሳተፍ ከፈለጉ አይገደዱም፡፡

በዚህ ጥናት ባለመሳተፍዎ ማንኛውንም አገልግሎት ከማግኘት አይከለከሉም፤ ነገር ግን የዚህ ጥናት ዓላማ ከተፈለገው ግብ እንዲደርስ የእርስዎ አስተዋፅኦ የላቀ ነው፡፡ በጥናቱ መሠረት የሚለዩ የተለያዩ ችግሮች ለመንግሥትና በሌሎች ድጋፍ ሰጪ ድርጅቶች ቀርበው እንዲፈቱ ይደረጋል፡፡

የምርምር ፕሮጀክቱ አላማ፤ የሳንባ ነቀርሳ በሽተኛ ቤተሰብ የቲቢ ምርመራ ለማድረግ እንቀፋቶችን ማሰስ የሚለውን ለማጥናት የተዘጋጀ ነው፡፡ ጥቅሞች:- እርስዎ ጥናቱ ላይ መሳተፍ አሁን ለግልዎ የገንዘብ ጥቅም ባይኖረውም፤የሚሰጡት መረጃ ግን ለጥናቱ መሳካት እና በጥናቱ ለሚለዩ ችግሮች መፍትሄ ሲሰጥ እረስዎ እና ሌሎች ሰዎች ተጠቃሚይሆናሉ።

ጉዳት:- እርስዎ በጥናቱ ላይ ስለተሳተፉ ከጊዜዎት በስተቀር የሚደርስብዎት ምንም ችግር የለም።

*ምስጢር ስለመጠበቅ:-* ለዚህ ጥናት የሚሰጡት መረጃ በሙሉ በምስጢራዊነት ይያዛል፡፡ ለዚህ ጥናት የሚሠበሰበው እርሰዎን የሚመለከት መረጃ በማህደር የሚቀመጥ ሲሆን ማህደሩም በስመዎ ሳይሆን በተለየ ኮድ ሲቀመጥ ኮዱ ከዋናው ተመራማሪ ውጭ ለማንም አይገለጽም፡፡

ጥናቱን በተመለከተ ሊብራራልዎት የሚፈልጉት ነገር ካለ መጠየቅ ይችላሉ። ለበለጠ መረጃ የጥናቱን ዋና መሪ በሚከተለዉ አድራሻ ማግኝት ይችላሉ። ስልክ ቁጥር ፡0928507199 ነው ጽፌ መስጠት እችላለሁ፡፡

የስምምነት ቅጽ ከላይ በዝርዝር የተሰጡትን መረጃዎችን ልረዳ በምችለዉ መልኩ በመረጃ ሰብሳቢዉ ተነቦልኛል፡፡ ስለሆነም በ ጥናቱ ላይ ለመሳተፍ ተስማምተዋል አልተስማሙም

ኤክሰ 2; ተስማምቻለሁ ፊርማ……..

ጠያቂ፤ እድሜ ስንት ነው?

ኤክስ 2፤እድሜ 38

ጾታ፤ ሴ

ጠያቂ፤የጋብቻ ሁኔታ ብትገልጭልኝ?

ኤክስ 2፤ባለትዳር

ጠያቂ፤ሃይማት

ኤክስ 2፤ክርስቲያን

ጠያቂ፤ የትምህርት ደረጃ እንዴት ነው

ኤክስ 2ደረጃ 4 ጤና ኤክስትንሽን

ጠያቂ፤እሽ ወርሃዊ ገቢ

ኤክስ 2 ፡6000 ብር

1.በጤና ኬላቹ የቲቢ ህክምና የሚከታተሉ ስንት ታካሚወች አሉ…3…………………በአንድ ቤት ውስጥ አብረዋቸው የሚኖሩ ሰወችንስ 9 ስለ ቲቢ ከ አስራ አምስት አመት በታች 3 ሁኔታቸው ለማዎቅ ምን አደረጋቹ………ምንም……….

ጠያቂ፤ሳንባ ቲቢ በሽተኛ ጋር በአንድ ቤት ውስጥ አብረው የሚኖሩ ሰወችን የቲቢ ሁኔታ በጤና ባለሙያወች አሰሳ እነዳይደረግ እንቅፋቶች ምንድን እንደሆኑ እስኪ ልምድሽን አጫውችኝ……………………………….

ኤክስ 2፤አመሰግናለው፡፡ እርስወ እንደገለጹት ያው ከጤና ስርአት ወይም ከመንግስት አንጻርም እና ከጤና ባለሙያወች ከማህበራዊ ና ባህላዊ አንጻር እንዳለ ሁኖ ከጤና ስርአት ወይም ከመንግስት አንጻር ሳብራራው ወይም ከመንግስት አንጻር ያለውን ጉድለት ስናይ፤ አንደኛ የቁጥጥር ማነስ ነው፡፡የቁጥጥር ማነስ ማለት ለምሳሌ ጤና ጣቢያወችና ጤና ኬላወች ትስስር አለመኖር በዋናነት ማለት ነው፡፡ከዛ ውጭ ስልጠና አለመሰጠት፤ጤና ኤክስቴንሽን እነደመሆኔ መጠን እኔ ስለቲቢ ምንነት የማወቀው እውቀት አለመኖሩ ነው፡፡በዋናነት ከመንግስት አንጻር ስናየው ፡፡ከዛ ውጭ የግብአት እጥረት፤ የግብአት እጥረት ስንል ያው እንትን የሪኤጀንት ወይም የሌላ ነገር አለመኖር ብለን ስናጠቃልለው፡፡ከዛ ውጭ ደግሞ ቶሎ ሂዶ ስንልካቸው ቶሎ ሂዶ አለመስተናገድ፡፡ቶሎ ተስተናግደው ያለመምጣት ፤የመጉላላት ችግር ይታያል፡፡ከመንግስት አንጻር ስናየው፡፡ከዛ ውጭ ግብረመልስ አለመስጠት፡፡ይህ ደካማ ጎን ነው፤ ይህ ጠንካራ ጎን ነው ተብሎ ተለይቶ አለመሰጠቱ ፤ እሱ እንደ ክፍተት እሚወሰድ ነውከመንግስት አንጻር ፡፡ከዛ ውጭ፤የሰው ሃይል እጥረት መኖር፡፡በዋናነት የሰው ሃይል እጥረት አለመሟላት፡፡እኔ ለምሳሌ አንድ ከመሆኔ አንጻር ከተለያዩ ክፍሎች መግባትና አንድን ስራ ለይቶ ትኩረት አለመስጠት፤ለምሳሌ እንደ ቲቢ ያለውን ማለት ነው፡፡ከዛ ውጭ የትራንስፖርት በጀት አለመኖር፡፡ቲቢ ታካሚውችን ሂደን ለማምጣት ስንል የትራንስፖርት ችግር መኖር፡፡ይህ አንዱ ክፍተት ነው፡፡እነዚህ የተዘረዘሩት ከመንግስት አንጻር ስናያቸው ያው ክፍተቱን ይፈጥሩታል ብለን ነው እናያቸዋለን ማለት ነው፡፡እንደ ጤና ኤክስቴንሽን አንጻር፡፡ከዛ ከጤና ባለሙያወች አንጻር ስንሄድ ፤ስለ ቲቢ ታካሚ ቤተሰብ እውቀት አለመኖሩ፡፡ምክንያቱም የጤና ባለሙያው ስለ ቲቢ ታካሚ ቤተሰቦች የቲቢ ሁኔታ አላቸው ወይስ የላቸውን የሚለውን አቅም አልነበረም ማለት ነው፡፡እሽ ከባለሙ አንጻር ስንወስደው ክፍትቱ፤ለምሳሌ ስለ ቲቢ ታካሚወች፤ ለምሳሌ በሽተኛው ነው እንጅ ምርመራ እንደሚወስድ እምናውቅ፤ቤተሰቡ እንደሚመረመር ስልጠና ክፍተት ስላለ፤የእውቀት ማነስ ስላለ እሱን ማድረግ አለመቻላችን፡፡ከዛ ውጭ ስልጠና ካለመውሰድ ጋር ተያይዞ የቁርጠኝነት ችግርም አለ፡፡የቁርጠኝነት ችግር ማለት፤በራስህ ተነሳሽነት እሰራዋለሁ ስራውን አቅዶ አለመነሳት፡፡ይህ በዋናነት እሚወሰድ ክፍተት ነው ብየነው እምወስደው፤ እኔ እንደ ጤና ኤክስቴንሽን ስራ አንጻር ፤ስራ ጫና መኖር፤በተለያዩ ክፍሎች መግባት ፡፡ እሱ አንድ እንደክፍትት ይፈጠራል ፡፡ምክንያቱም ከእናቶች ከህጻናት ከተለያዩ ክፍሎች አንድ ሰው ፡፡የስራ ሎድ ሲበዛበት ያንን ስራ ወደ ኋላ የመጣል ነገር አለ፡፡ለምሳሌ ቲቢን ማለት ነው፡፡ስለዚህ ይህ አንዱ ክፍተት ነው ማለት ነው፡፡የሰው ሃይል ማነስ ቅድም ስለገለጽኩት ማለት ነው፡፡እሱ ዋና ክፍተት ነው ማለት ነው፡፡የቲቢ በሽተኞች ቅድም ገለጨዋለው የቲቢ ጤና ዳሰሳ አለማድረግ ፤ምክንቱም እኛ በሽተኛውን ነው እንጅ የእውቀትም ማነስ ስላለ ቤተሰቡን አለማድረግ፡፡ይህ ቤተሰቡን ሂደን አሰስ ማድረግ ነበረብን ግን እውቀት ማነስ ስላለ እሱን አላደረግንም፡፡ሌላው ከቤተሰብ ላይ ስንነሳ ፤አንደኛ እነሱ እውቅና የላቸውም፡፡እውቅና አልሰጠናቸውም ማለት ነው፡፡ቤተሰቦቻቸውን ፡ በመጀመሪያ እነሱ ቅድሚያ ለስራቸው እንጅ ቁርጠኝነት የላቸውም፡፡ለበሽታው ሂዶ ለመታየት ቁርጠኝነት የላቸውም፡፡ ለስራቸው ነው ቅድሚያ መስጠት እምፈልጉ ማለት ነው፡፡ ከዛ ውጭ ከማህበራዊና ባህላዊ ስንነሳ፤ርቀት አለው፡፡ቅድም እንደጠቀስኩት ትራንስፖርት ችግር አለ ብየ ጠቅሸው ነበርና፡፡ርቀት ስላለ ወጣ ገባ ነው፡፡ ቢጓዙም ሶስት ሰአት ከዛም በላይ ነው፡፡ወንዝ ነው፡፡ ስለዚህ ይህ አንድ ፈታኝ ነገር ነው ማለት ነው፡፡ማህበራዊ ስንነሳ፤በማህበራዊ ላይ ይገለላሉ ሰወች፡፡ ምክንያቱም ቅድም እንዳነሳውት ለስራቸው እንጅ እነሱ ሂደው ጤናቸው ትኩረት ስለለላቸው ፤አንድ ሰው ቲቢ አለበት ከአንድ ቤተሰብ ከተባለ ፤የመገለል ነገር አለ ማለት ነው፡፡ከኢኮኖሚ ስንነሳ፡ ቅድም እንዳልኩት በጣም ሩቅ ስለሆነ በሞተር መምጣ ይቸገራሉ፡፡ትራንስፖርትም ላይኖራቸው ይችላል፡፡ይህ ፈተኝ ነው ማለት ነው፡፡ቅድም ብየዋለው መገለል ከእድር ከምን ከሰምበቴ፤የመገለል ነገር አለ፡፡ያው እንግዲህ ባጭሩ ከጤና ኤክስቴንሽን አንጻር ብዙም ባላብራራው ያለኝ ነገር ይህ ነው፡፡አመሰግናለው

ጠያቂ፤እናንተ ሂዳችሁ እንዳትመረምሩ እንቅፋት የሆነባችሁ ምንድን ነው?

ኤክስቴንሽን 2፤ ቅድም ለመግለጽ እንደሞከርኩት፤አንደኛ ቲቢ ታካሚወች እንዲመጡም አይገደዱም አንተ እንዳልከው፤ ባሉበት ሂደን እንድናቸው ነው እሚፈለገው፡፡ያው የትራንስፖርት ችግርም እጥረትም አለ፡፡ከዛ ውጭ የኛ የግንዛቤ ክፍተትም አለ ብየ ነው እምወስደው፡፡

ጠያቂ፤ መንግስት የሚያደርግላችሁ ድጋፍ ምንድን ነው?ስለ ቲቢ ታካሚ ቤተሰብ እንደሚመረመሩ ምን ድጋፍ አደረገላችሁ?

ኤክስቴንሽን 2፤ ቅድም ለመግለጽ ሞክሪያለሁ፤ ያው ከመንግስት አንጻር ፤ይህ የቲቢ በሽታን ወደ ኋላ የመተው፡ቅድሚያ ለእናቶች ለህጻናት የመስጠት ነገር ስላል፤ ወደ ኋላ የመጣል፤አንድን ተግባር ከሌላው አስበልጦ የማየት ነገር አለ ማለት ነው፡፡ከመንግስት አንጣር ስናየው፤የክትትልና ቁጥጥር ማነስ ነገር አለ፡፡ምክንያቱም ስለ ቲቢ ሳይሆን ስለ ህጻናት እና ስለ እናቶች ቅድሚያ ሰተን በመስራታችን ፤ይህ ክፍተት አለ ብየ ነው እምወስደው፡፡

ጠያቂ፤በእናንተና በጤና ጣቢያው መካከል ያለው ግንኙነት ምን ይመስላል?

ኤክስቴንሽን 2፤ እሽ በጤና ጣቢያውና ቅድም ለመግለጽ እንደሞከርኩት በጤና ጣቢያውና በጠና ኬላው መካከል ያለው ትስስር የላላ ነው፡፡ምክንያቱም ቲቢ የተረሳ ተግባር ነው ፡፡አንድ ቤተሰብ ውስጥ ታካሚው ኑሮ ቤተሰብ ቶሎ እንዲታይ ታካሚው ቶሎ እንዲታከም የማድረግ ሁኔታ የለም፡፡ቅድም እንዳብራራውት ፤ቅድሚያ ህጻናትን እናቶችን፤ሌሎችን ተግባር የማስቀደም፡ነገር ስላለ ያው ትስስራችን የላላ ነው፡፡

ጠያቂ፤ ቤተሰብ እንደሚመረመሩ ጥንቃቄ ማድረግ እንዳለባቸው የሰጣችሁት ግንዛቤ ምን ይመስላል?ምን አደረጋችሁ?

ኤክስቴንሽን 2፤እኛ ያደረግነው ነገር የለም፡፡ቅድም ለመግለጽ እንደሞከርኩት፤ እኛ ግንዛቤ አልፈጠርንም፡፡የእውቀት ክፍተት አለ ብየ ገልጨዋለው፡፡አንድ በሽተኛ መጥቶ እንኳ በሽታ ቢገኝለት እንደሚታከም እንኳ የማብራራት አቅሙ የለንም፡፡ምክንያቱም የክህሎት ጋፕ አለ፡፡የክህሎት ክፍትት ስላለ ፤እሱም መመርመር እንዳለበት እሱም መታከም እንዳለበት ፤ቤተሰቡም መታከም እንዳከለበት የሰጠነው ነገር የለም፡፡

ጠያቂ፤ ከማህበራዊና ባህላዊ አንጻር ወደ ባህላዊ ህክምና የመሄድ ነገር አለ ይባላል ይህ እንዴት ነው?

ኤክስቴንሽን 2፤አንዳንዴ ያው እንግዲህ እኛ ትምርቱን አልሰጠንም በየ ለመግለጽ ሞክሪያለው፡፡ስለዚህ ማህበረሰቡም እውቅና ስለለለው ፤በሽታው አንዳንዴ በሽታወች ወደ ባህል የመግፋት ነገር አለ፡፡እና የቤት ህመም ነው ምናምን፤እሚሉት ነገር ስላለ፤ማህበረሰቡ ያው ፡በባህል ነው እሚጠቀም አብዛኛውን ጊዜ ማለት ነው፡፡

ጠያቂ፤ ማጠቃለያ…በመጨረሻ እሚጨርሱት ካለ?

ኤክስቴንሽን 2፤ያው ፈታኝ ተብለው የተቀመጡ ነገሮች፡አለ እምንላቸው ያው፡ ቅድም ለመግለጽ እንደሞከርኩት ፤ያው አሁንም ችግሮችም እነዲባባሱ የሚያደርጉት፤ባህል የቤት ህመም ነው ብሎ ማመን፤የግንዛቤ ክፍተት መኖር፡ይህ ዋና ችግር ነው ብየ ነው እምወስደው፡፡ህብረተሰቤ ይህን እውቅና አለመኖር፡፡በጤና ተቋም ታክሞ መዳን አለመቻሉ፤እኛም ግንዛቤ አለመፍጠራችን፤ህብረተሰቡም አለማወቁ ሰፊ ክፍትት አለ፡ብየ ነው እማምነው፡ያው እነዚህ እነዚህ ችግሮች ካልተቀረፉ፤ ችግሩ እየተባባሱ እንደሚሄዱ፤ከመንግስትም አንጻር ፡የጠቀስኳቸው ነገሮችም አሉ፡ከባለሙያም አንጻር ፡የጠቀስኳቸው አሉ፤እና ከማህበራዊና ከኢኪኖሚያዊ እሚለው ነገር ፤እሚቀረፍ ባለሙያውም እውቅና ኑሮት፤ማህበረሰቡም እውቅና ካልተፈጠረለት፤ችግሮች ይባባሳሉ፡፡ባጭር ጊዜ መቀረፍ አለበት ብየ ነው እማምን፡፡ጨርሻለው አመሰግናለው፡፡

ጠያቂ፤እኔም አመሰግናለው፡፡

Questioner: Tell me about your experience, what are the obstacles to health experts investigating the TB status of people who live in the same house with a TB patient?

X2: Thank you. As she explained, the same is true from the perspective of the health system or the government and from the social and cultural perspective of the health professionals. The first is the lack of support. Lack of support means, for example, the lack of connection between health centers and health centers. Apart from that, lack of training, as I am a health extension worker, there is lack of knowledge about the nature of TB. Mainly from the government's point of view. There is also lack of resources. When we say lack of resources, we mean the absence of reagents or other things. Apart from that, when we go and send them quickly, they are not handled quickly. Other than that, not giving feedback. Not identified as This is a weakness, this is a strong point not given feedback; considered as a gap from the point of view of the government.. These are listed from the government's point of view. It means that we see them as creating the gap. Apart from that, there is a shortage of human resources. For example, because I am one, entering from different departments and not paying particular attention to one work, for example, it means the one like TB. Apart from that, there is no transportation budget. There is a transportation problem when we want to go and bring our TB patients. This is one of the gaps. These are barriers listed from the government's point of view; we see them as creating the gap. And then when we go from the point of view of health professionals, the lack of knowledge about the TB patient's family screening. The lack of knowledge, because the health professional did not have trained to know whether the families of TB patients have TB status or not and screen them. For example, we know that it is the patient who is investigated, but not have awareness about contact investigation, because there is a gap in training for the family to be examined, because there is a lack of knowledge, we are unable to do it. Besides, there is also a commitment problem related to not taking training. Commitment problem mean not planing, I can do this work by my self not intersted. Apart from that, there is work over load, working in different department. For example In terms of my work as a single health extension worker, there is a lot of work, and I have to go to different departments like under five, vaccination and maternity. It created as an opening. Because one person from different departments from mothers to children. When the work load is heavy, there is something to put that work behind. For example, TB. So this is one of the gaps. It is because of the lack of manpower that I mentioned earlier. It means that it is the main gap. We explained earlier that we do not do a TB screening for TB patients contact, because we care about the patient, but because there is a lack of knowledge, we did not do it for the family. We shall screen contacts. But we did not screened them, because lack of awareness of health professional. The other, when we see from the point of contacts and the index case, there is lack of awareness, and we didn’t creat awareness for them. First of all, they are not committed to be screened, priority for their work. It means they don't want to go health institutions and be screened, give priority to their work. Apart from that, when we start from social and cultural aspects, there is distance. As I mentioned earlier, I mentioned that there is a problem with transportation. Even if they travel three hours is more than that. It is a river. So this is a challenging thing. When we start socializing, people are isolated on social; whene there is a pulmunary TB patient in a familly, the society discriminat them. As I said before contacts and the index cases are discriminated from social activities like edir and senbete. Because as I mentioned earlier, instead of their health, they focus on their work.from the economic view I mentioned before that there distant and difficult for motor transportation, there is lack of money for transport in low economic status. In short, this is what I have; not explained much in terms of health extension knowledge. Thank you.

Questioner: What is preventing you from going and investigating?

Extension 2; as I tried to explain earlier, TB patients are not forced to come as you said. We need to go and save them where they are. There is also a lack of transportation. Apart from that, there is also a gap in our understanding.

Probe; what kind of support does the government give you?

Extension 2; I tried to explain it earlier. From the point of view of the government, this is about leaving TB disease behind, giving priority to mothers and children. It means that there is something to put it behind, to see one activity as better than another. When we see it from the government, there is a lack of supervision and control. Because we have prioritized children and mothers, not TB.

Questioner: What is the relationship between you and the health center?

Extension 2; as I tried to explain earlier, the connection between the health center and health post is loose. Because TB is a forgotten activity. There is no way to make the patient live in a family so that the family can be seen quickly and the patient can be treated quickly. Because there is something, our bond is loose.

Probing; what is your impression that the family should be careful about being tested? What did you do?

Extension 2: Nothing we did. As I tried to explain earlier; we have not created an understanding. I explained to him that there is a knowledge gap. Even if a patient comes and is diagnosed with a disease, we do not have the ability to explain that he will be treated because there is a skill gap.

Probong; it is said that there is something to go to traditional medicine from a social and cultural point of view. How is this?

Extension 2; Sometimes he did not give us the lesson. I have tried to express it in every way. There is something about the disease sometimes pushing the disease into the culture. And we believe it is home sickness, because there is something they say, the society is the same: it is used by the culture most of the time.

Questioner. Summary...if you're going to finish it at the end?

Extension 2: The same things that are set as a challenge: I believe them, as I tried to explain before, and the problems are still getting worse, believing that culture is a home sickness, having a gap in awareness, I mean to say that this is the main problem. My society does not recognize this. There is a wide gap in the fact that an institution cannot be treated and cured, and that we do not create awareness, and that the society does not know. As the problem is getting worse, there are things that I have mentioned from the government's point of view, there are also things that I have mentioned from the professional's point of view, and from the social and economic point of view, the problems that need to be solved are the recognition of the professional, and if the society does not recognize it, the problems will get worse.

Questioner: Thank you too.

3

ኤክስ 3

ጠያቂ፤ ከሳንባ ቲቢ በሽተኛ ጋር በአንድ ቤት ውስጥ አብረው የሚኖሩ ሰወችን የቲቢ ሁኔታ በጤና ባለሙያወች አሰሳ እነዳይደረግ እንቅፋቶች ምንድን እንደሆኑ እስኪ ልምድሽን አጫውችኝ……………………………….

ኤክስ 3፤ ያው ችግሩን እንትን ያልነው፤ አጠቃላይ በቃ በማህበረሰቡ ዘንድ የግንዛቤ ችግር አለ፡፡ የግንዛቤ ችግርና ከስራ አኳያ ደግሞ ቅድሚ ለስራቸው ነው እሚሰጡት፡፡ያው ገጠር እንደመሆኑ አንጻር ቅድሚያ ለስራቸው ስለሚሰጡ ነው፡፡ሌላ ምንድን ነው ተስፋ መቁረጥ አለ፡፡አለብህ ሲባል በቃ ግድ አሁን እነሱ በግድ ነው እሚመጡት፤አለብህ ከተባለ ነገ እሞት ይሆን እያለ ተስፋ መቁረጥ አላቸው፡፡ቀዉርጠኝነትና ተነሳሽነት ትኩረት አለመስጠታቸው ነው፡፡እንጅ ሌላ ችግር የለም፡፡ለወደፊቱ ግን ይህንን በትኩረት በቃ ለመስጠት ያው ግምገማው ላይ አስበናል፡፡ሌላ ከባለሙያ ከጤና ተቋሙ በተለይ ግብረመልስ አለመስጠት፡፡ስለጠናም አለመሰጠቱ፡፡ግብረመልስም አለመሰጠቱ፡፡ግምገማና ክትትሉ ደግሞ አነስተኛ መሆን፡፡ምንድን ነው ስልጠና አለመሰጠቱ ስል በትኩረት ተቋሙ አስቦ ከመንግስትም አንጻር በቃ ስልጠና አለመሰጠቱ፡፡ከተገኘባቸው አንዳንድ ባለሙያ ለምሳሌ ከተገኘባቸው እነዚያን ክትትል እንጅ፤ በጎንዮሽ ያኛው አልተመረመረም እሚያስተላልፍ መስሎ አልተሰጣቸውም፡፡ምክንያቱም ስልጠና ባግባቡ አልተሰጠም፡፡ከዛ ግምገማና ክትትሉ እራሱ አሁን በኛ በየሶስት ወሩ በየ ሩብ አመቱ ነበር ግምገማ ክትትል እሚደረግ ማለት ነው፡፡ግን ግምገማና ክትትሉ ትኩረት ማነሱ ነው፡፡አሁን ለምሳሌ የት ደረሳችሁ ተብሎ ግምገማና ክትትል ቢያንስም እንኳ ግብረመልስ በኛ ይሰጥ ነበር፡፡አሁን ግን ግብረመልስ አልተሰጠም፡፡ ግብረመልስ አለመሰጠቱ ነው ትኩረት ያልተሠጠበት ምክንት ማለት ነው፡፡ሌላ ያው ከማህረሰቡ አኳ ያው የኢኮኖሚ እጥረት መሆኑ ነው፡፡ችግርም አለባቸው ለምሳሌ ቦታ ርቀት፤ አሁን እነሱ የማይመጡበት ምክንያት ቢያውቁም እንኳን በጣም ከርቀት አንጻር፤ ለምሳሌ እንደልብ ሃብት ቢኖር በሞተር እንኳን ይሄዳሉ፡፤ያንን አስር ቤተሰብ እንዴት አድርጎ አስሩን ቤተሰብ ሁል ፡ያው መኪና ስለለለ ተስፋ ይቆርጣሉ፤እንዴት አድርጌ ተመላልሰን እንደዚህ ብለው ፤በተለይም ደግሞ ውጣ ውረድ እንደመሆኑ አንጻር ፡እንደገጠር አንጻር በቃ የኢኮኖሚ ችግር ስላለባቸው ይመስለኛል ትኩረት ያለሰጡበት ማለት ነው፡፡ሌላ ማግለል፤ አሁን ለምሳሌ ቲቢ ተብሎ በቃ ትኩረት ያልተሰጠበት እንደኛ ማህበረሰብ ገና እንትን እሚለው ማለት ነው ማግለል አለ፡፡አሁን ቲቢ አለበት ከተባለ በቃ መድሃቱ እራሱ በግድ ነው፤ እኛ ማልደው ሌሊት መተው ነው እንጅ ማግለልም አለ፡፡መገለል አለባቸው፡፡ አሁን እነሱ ቲቢ ከያዛቸው ማለት የሆነ ጫና እራሳቸው አምጥተው እንትን እሚሉት እሚመስላቸው አሉ፡፡ነገር ግን ይህንን ባለማወቃቸው ነው፡፡ደግሞ እማይድኑ ይመስላቸውል ፡፡ተስፋ ስለሚቆርጡና እነሱን ብቻ እንደዚህ ሁነናል ሌሎችስ ብለው እንዲያው እነሱ እራሱ መድሃታቸውን ሲወስዱ በሌሊት መተው ነው እሚወስዱ፡፡መገለል አለባቸው ማለት ነው፡፡ስለዚህ የመገለል ችግር ስላለባቸው ይህን ይህን ግምገማ ለይተናል፡፡እንግዲህ ለወደፊቱ ትኩረት ሰተን እንሰራበት ሁኔታ ነው እሚመስለው፡፡ሌላ ከጤና ባለሙያ አንጻር በጣም ችግር የሆነበት ለምሳሌ እኔ ብቻየን ነኝ እምሰራው፤ የተለያየ ክትባት አለ፤የእናቶች ልየታ አለ፤ፓኬጅ አለ፤እለት ከእለት በጣም መባዘን ነው እሚሆን ፡፡ሁሉን ነገር ለቅመን ለመስራት ትንሽ ያዳግተናል፡፡ምክንቱም ከቦታው አንጻር ፤አንድ በአንድ በቃ ክትባቱ ተበታትኖ ሶስት አውድ እለት ነው ያለን ፡፡ የሶስቱ አውደእለት ደግሞ ሲያቋርጥ ሁልጊዜም ቢሆን ያንን እለት እለት ህጻናትን ስለምንከታተል፡፡በተጨማሪም ከአምስት አመት በታች የሆድ ትላትል አልቤንዳዞል ወይም ደግሞ ቫይታሚን ኤ ስንሰጥ ያንን በብዛት ስንሰጥ ስለሆነ በቃ ከድካም አንጻር በጣም ብዙ ስራ ስለምንሰራ ለዛም ነው እኛ ትኩረት ያልሰጠንበት፡፡በተለየ የስራ ጫና አለብን ፡፡የስራ ጫና አለብን፡፡ገጠር ነው እምንሰራ፡፡ሁልጊዜ ተመላልሰን ነው እምንሰራ ፤ለምሳሌ ፓኬጅ እንላለን፡፡እናቶችን ልየታ ነፍሰ ጡር ልየታ እንላለን፤ በዛ ላይ የእናቶች ኮንፈረንሰ ካለመጣች ምን ሆንሽ እንላለን፡፡በዛ ላይ ደግሞ ህጻናት ያቋረጠ ዕንመርጣለን፡፡በጣም የባዘነ ስራ በጣም ብዙ ስራ አለብንና ብቻየን እንደመሆኔ ብቻየን ስለሆንኩኝ ፤ለዛ ነው ትኩረት ያልተሰጠበት፡፡

ጠያቂ፡ ግብአትና የሰው ሃይል አካባቢ ምን ችገር አለ እምትጨመሪው ካ በናጸ በኩል፡፡

ኤክስ 3፡ያው የኛ ከግብአት አንጻር በአግባቡ መድሃኒቱን በአግባቡ ሁልጊዜ ይደርሳል ማለት አይቻልም፡፡ለምን ከመንግስት አንጻርም የመድሃኒት እጽረት አለ ነው እምለው፡፡ከባለሙያ አንጻር የባለሙያ አጥረት የሰው ሃይል በጣም ችግር አለባቸው ማለት ነው፡፡ጤና ጣቢያም ቢሆን የሰው ሃይል ችግር ስላለባቸው ይመስለኛል ፤የታመሙትን አንድ በአንድ ክትትል አርጎ መጥቶ ከማከም ውጭ ፤ቅድሚያ መተው ከኛ ብለው ያው ክትትልና ድጋፉ አነስተና ነው የሆነ ማለት ነው ምክንያቱም፡ባለሙያ ሰው ሃይል እጥረት ስላለ፡፡በተለይ ደግሞ ከኛ ቀበሌ አንጻር ያው ብቻየን በመሆኔ በጣም ያው የሰው ሃይል እጥረት አለ፡፡ይህንንነ ደግሞ መንግስት አይቶ እለት እስከ እለት ክትትል ቢያደርግልንና በደንብ በብዛት ከኛም ቢያንስ አንዳንድ ቀበሌወች ላይ ሶስት ጤና ኤክስቴንሽን አለ፡፤እኔ ግን ብቻየን ነኝ ያለሁት ፡፡ይህንን ግን በትኩረት ቢያውና መንግስት በደንብ በአንድ በአንድ ቢሆን የተሻለ ስራ ማህበረሰቡን እናገለግላለን ብየ አስባለው፡፡ ከግብአት አንጻርና ያንንም ቢከታተል የበለጠ የተሸለ ነው ብየ አስባለው፡፡

ጠያቂ፤ እናንተ ሂዳችሁ ስክሪን እንደታደርጉ የገጠማችሁ ምንድን ነው፡፡

ኤክስ 3፡ያው እኛ በተቻለን መጠን ሂደን እንናገራለን ፡፡አንደኛ ሂደን መናገርና መምከር ነው እንጅ እንደጤና ኤክስቴንሽን ስክሪን ይደረግ አልተባለም፡፡እስካሁን አላደረግንም ፡፤ጤና ኤክስቴንሽን ሁኖ መቸም ስክሪን እሚያረግ ነገር የለም፡፡ቅድም እንደነገርሁህ ማለት ነው የባለሙያ እጥረት ስላለ እነሱም ተከታትለው ሂደው ቢያንስ ከቤትም ቢሆን ፤የሆነ የተሸለ ነገር መምከር አልመከሯቸውም ፡፡እኛ ግን በትኩረት ሂደን እንመክራለን፡፡ለወደፊት የሚሀነው ነገር እንግዲህ በተቻለ መጠን አግባብተንም ቢሆን እያረፉ ምንም ትራንስፖርት ቢያጡ ተራ በተራ ቀስ እያሉ እንዲመጡና ከሳምንት ባላነሰ ጊዜ በቃ ሂደን በደንብ ትኩረት ሰተን መክረን ሂደው እንኳን ቢያንስ ከጤና ተቋሙ ላይ ሂደው እንዲታከሙ ወይም ስክሪን እንዲደረጉ እናደርጋለን፡፡

ጠያቂ፤ ከባህላዊ አንጻር ምን ችግር አለ እንዳየመረመሩ የሚያደርግ ከባህላዊ አንጻር ያለውን ችግር ቢገልጹልኝነ

ኤክስቴንሽን 3፤ ከባህላዊ አንጻር እንግዲህ በጣም ያም ነው ትኩረት ፍጓቸው፤ቢያንስ የኢኮኖሚ ችግሩ እንደተጠበቀ ሁኖ ማለት ነው ባህላዊም በደንብ አልተቀረፈም፡ እንደኛ ገጠር ፤ገጠር እስከሆነ ድረስ እንዲያውም እሚገርመው ነገር እነዚህን እድላም ነን በስራተ በግደ ነው በዚህ ሰአት እግዚአብሄር ይመስገን የተገኘባቸው እንጅ አጉል በጸበል ነው እሚሄዱት፤ሌላው ደግሞ ያው ያምናሉ ፤በደንብ በትኩረት ሂደው ደግሞ የባህል ህክምና ያደርጋሉ ፡፡በግድ መቸም ተመስገን ብሎ ብሎ በግድ ሂደን ያው ሳል ሰለሚበዘባቸው በታም ሳል ስለሚበዘባቸው ደግመን ደጋግሜ ተናግረን ያው እነዚህም የመጡት፤የሚገርመው

ነገር ቲቢ ነው አሉ ሲባል ትኩረት አልተሰጠውም፡፡ትኩረት ትንሽ ይቀረዋል ፡፡ህብረተሰቡ አልነቃም ዝም ብሎ በቃ ገዳይ በሽታ ወይም ደግሞ እራሳቸው እንዳመጡት ነገር ፤እንደማግለል ሲያቸው ነው ፤አሁን ያንን ቤተሰብ ወደ ኋላ ያላቸው ቢያንስ ቢተይባቸው እኛም ተገለናል ብልው ሁለታችን እንኳ ተገለናል ደግሞ ሌሎችን ያለኝ አለ ፡፡በተለይ ከአንዱ ሂጀ ነበር እኔ እና፤በትኩረት ምንም እንደሌለ ሲታከሙ መዳን እንደሚችሉ ነው በተቻለ መጠን አገዕም ቢኖረኝ በተለይ ከጤና ተቋም አንድ ሰው አብሮኝ ቢሄድ ያንን እንነግራለን እንጅ ኋላ ቀር ነው ያው ህብረተሰቡ ብዙም በቲቢ ላይ ትንሽ ይቀረዋል፡፡

ጠያቂ፣ ማጠቃለያ …በመጨረሻ እምትጨመሩት ነገር ካለ የቀረ ነገር አለ እምትይወ ካለ፤

ኤክስ 3፤ያው ይህንን ከጠቀስኩት እውነት ይህንን ሲሆንም ሲሆንም መቶ ፐርሰንት ካለዚያ ሃምሳው ፐርሰንት እንኳ ቢቀረፍ የተሸለ ንወ፡፡ያው እንግዲህ ወየው ይህ ከተገለጸ በትኩረት መንግስት ከሰጠ የተሻለ አዲስ ነገር እኒኳን ከዛ ውጭ ሌላ ነገር የለኝም እኔ

ጠያቂ፤አመሰግናለው

Questioner. Tell me about your experience.

X 3; what is the problem? There is a general lack of awareness among the community. In terms of awareness problems and work, they give priority to their work. Since it is a rural area, they give priority to their work. What else is there to give up, if they know there is TB; they will assume I will die tomorrow. They don't pay attention for screening and have no commitment and initiative .there is no other problem. But for the future, we are thinking of paying attention to this in the same evaluation. Another from the health care worker and from the health institution, especially, is not giving feedback, the lack of training, the lack of feedback, and the lack of evaluation and monitoring. Evaluation and monitoring should be minimal. When I say that training is not provided, from the point of view of the institution and the government, the lack of sufficient training.the institution should focus on the lack of training from the government's point of view. On the other hand, when there is TB case only follow that case but don’t care for the contacts whether he transfer or not. Because training was not given properly. Then the evaluation and monitoring itself was done every three months and every quarter. But the evaluation and monitoring is to draw attention. There is no meeting to evalute whwre we are. Feedback used to be given by us, but now no feedback is given. The lack of feedback is the reason why it was not given attention. Another thing is the lack of economy in the community. They also have problems such as distance. Now even if they know the reason why they don't come, even remotely; For example, if they have the enough money, they will even go by motorbike. How can they do that ten families, they give up because the ten families always have the same car, how can we go back like this? Especially in terms of ups and downs, I think it is because they have economic problems that they have not paid attention to. Another is discrimination, now, for example, like in our society, where TB is not given much attention, there an isolation. Now if it is said that some one has TB; he is afraid of taking drugs. TB patients come early at night, but there is isolation. They should be isolated. Now they think that if they get TB, they bring some kind of pressure on themselves and say, 'What's wrong? But because they don't know this, they think that they won't be cured. Because they give up hope and let others say that they are the only ones like this. Therefore, we identified this assessment because they have problems of isolation. So, it seems that we should pay attention to it in the future. Another problem from a health professional's point of view, for example, I work alone; there are different vaccines, there is maternal care, there is a package, it will be multiplied every day. It is a bit difficult for us to do everything in order. And there is three-day schedule of vaccination, we always follow the children. In addition, when we give albendazole or vitamin A for stomach worms under the age of five, we give it in large quantities. We have workload. It's rural, we work. In addition, if the mother's conference does not come, we will say what happened to you. In addition, we choose children who have dropped out. We have a lot of work to do, because I am alone because I am alone, that's why it is not paid attention to.

Probing: What is the problem in terms of resources and human resources?

X3: In terms of resources, it is impossible to say that we always get the medicine properly. Why is, there a shortage of medicines from the government's point of view? From a professional point of view, the lack of professional staff means that they have a serious problem. I think that the health center also has a staff problem. Apart from following up and treating the sick one by one, they say that they give up the priority and the support from us is less because there is a shortage of professional manpower.Especially in terms of our kebele, because I am working alone, there is a shortage of manpower. If the government sees this and monitors us day by day, there are three health extensions for at least some of our kebeles,but I am alone. I think that if the government works well one by one, we will serve the society better. I think it would be better if he followed up on that in terms of input.

Probe: What did you experience when you went and screened?

X3: Yes, we go and talk as much as we can. First, we go and talk and advise, but it is not said health extension sall screen. We have not done it yet. There is no health extension who screen at all. As I told you before, there is a lack of expertise, so they should not follow up and go at least from home. If so, I don't advise them to recommend something better. But we advise them to go with attention. The best thing for the future is that if they miss any transport while resting, they should come slowly one by one, and in less than a week, just go and pay attention and advice them to go and at least go to the health facility to be treated Or we'll have them screened.

Probing;. What is wrong with culture? If you explain to me the problem from a cultural point of view that keeps them from investigating?

Extension 3; from a cultural point of view, it is very important to ignore them. Culturally, it is not well addressed. As long as it is rural like us, the surprising thing is that we have these opportunities because we have to work. At this time, thanks be to God, they go with great prayer, and others believe the same thing, they go carefully and do cultural treatment. But when they said it was TB, they didn't pay attention to it. The community didn't wake up. It was just a deadly disease or something that they brought. Especially from one of them, I was told that there is nothing that can be cured when treated.

Interviewer, summary...Finally, if there is anything you would like to add, there is something left over;

X 3: Yes, if I mention this, the truth is this, and it would be better if 100 percent of that was removed, even 50 percent.

**4**

**ኤክስ 4**

ጠያቂ፤ ከሳንባ ቲቢ በሽተኛ ጋር በአንድ ቤት ውስጥ አብረው የሚኖሩ ሰወችን የቲቢ ሁኔታ በጤና ባለሙያወች አሰሳ እነዳይደረግ እንቅፋቶች ምንድን እንደሆኑ እስኪ ልምድሽን አጫውችኝ……………………………….

ኤክስ 4፤ እንዳንመረምር የሚያደርጉ ብዙ ችግሮች አሉ፡፡በእርግጥ አሁን እና እንደሚመረመርም በጣም ባናውቀውም ግልጽ ሁነን ፤እንደሚመረመር የተወሰነም ቢሆን እናወቀዋለን፤ ግን ግልጽ ሁነን ለማስመርመር ደግሞ እኛ የተለያዩ ችግሮች አሉ፡፡ለምሳሌ ሲነሳ ከመንግስታዊ ስታየው ፤ለማስመርመር እኛም ግልጽ የሆነ ስልጠና መውሰድ አለበት፡፡ለነሱም ቢሆን ድጋፍ ለማድረግ እኛ ውስጡን ገባ ብለን ማወቅ አለብን፡፡ለሰወች ድጋፍ ለማድረግ ይህን ይህን አድርጉ ብለን ለመምከር፤ስልጠናም መሰጠት አለበት ፤ እነሱንም እኛ መቀስቀስ አለብን፡፡እኛ ስልጠና ሲሰጠን ነው እነሱን መቀስቀስም ማስተማርም እምንችል፡፡ማህበረሰቡም ሊመረመር እሚችልም እኛ ባስረዳነው መልክ ነው እንጅ ዝም ብለን አይደለም፡፡ እና በመንግስት በኩል ስለቲቢ እሚሰጠው አመለካከት ይህን ያህል አይደለም፡፡ልክ አሁን እንደምታየው የእናቶችና የህጻናት ተግባራት ሌሎች ተግባራችና የቲቢ ተግባራ እኩል አይደለም እየታየ ያለ ፡፡ምክንያቱም ቅድሚያ እሚሰጥ የእናቶች ነው፡፡ቲቢ ተጠርጣሪ ካለ ለዩ ይባላል እንጅ ፤ቲቢ ምንድን ነው ምናምን የሚለውን ነገር በደንብ የብቻው ተሰቶ ስለጠና እየተሰጠ አይደለም ፡፡ከዛ ውጭ ደግሞ ቤተክርስቲያን ሂደን ሳል ያለባችሁ ተመርመሩ ነው እንጅ እምንል ፡፡ሌላውን ምን አይነት ሳል ምናምን እሚለውንም ሁሉን እሚያውቅ ጤና ኤክስቴንሽን የለም፡፡በደንብ አርጎ ማለት ነው፡፡እንደዛ ስለሆነ በመንግስትም በኩል ያለው ነገር ቀዝቀዝ ያለ ስለሆነ ትኩረትም ስለማይሰጠው እኛም ብዙም እውቅናም ስለለለን ፤መመርመር አለባቸው ሲሉን ከዛ ከጤና ጣቢያው መመርመር አለባችሁ ብለን እንነግራቸዋለን እንጅ ይህን ይህን ችግር ስላለ ብንላቸው ሰወችም ይሄዳሉ፡፡ግን ደግሞ እኛ ብዙም እውቅናው ስለለለን ሂዳችሁ ተመርመሩ እንላለን እነሱም አልሄዱም ፤ምንም ምረመራም አላገኙም ፡፡ከዛ ውጭ በኛ በኩልም በሙያተኛ በኩልም ያሉ አንዳንድ ክፍተቶች አሉ፡፡ያው እነዛ በኛ በኩል ያሉ ክፍተቶች እንግዲህ ከዛ ወደ ጤና ጣቢያ ነው ላኩ እምንባል እነዚህን የበሽተኛወቅን ቤተሰቦች እንዲመረመሩ ፤እና እኛም በደንብ ክፋቱን ጉዳቱን ስለማናውቀው ጠንከር አርገንም ውደዛም ብዙም አንልክም፡፡ንቁ የሆነ ተሳትፎም የለንም ያንን ለማድረግ እንደዛ ስለሆነ ይህ ይህ አንድ የሙያተኛ ችግርም አለ፡፡ከዛ ውጭ ደግሞ ከዛም ሂደው ያው እኛ ጠየና ኤክሰቴንሽን ብቻ ሳንሆን እዛው መርማሪውችም ቢሆን ትኩረት ሰተን እሚላኩትንም ቢሆን ትኩረት ሰተው ለቲቢ ብለን የላክናቸውን ትኩረት ሰተው እንትን አይሏቸውም አንድ ጊዜ እዛ አንድ ጊዜ እዛ ሲሏቸው ምርመራውን ለማድረግም እነሱ ሲማረሩ ተመልሰውም ይመጣሉ፤እና ይህ ደግሞ ትኩረት ባለመሰጠቱ ነው ከባለሙያውም አከኳያ ከጤና ጣቢያውም አኳያ እንደዚህ አይነት ነገር ሰስላለ ፤እነሱ እነሱ የሙያተኛውን ችግርም ይገልጻሉ፡፡ከዛ ውጭ ደግሞ ቅድሚያ አለመስጠት ነው፡፡ለነዚህ የቲቢ ታካሚወች ቅድሚያ አይሰጥም፡፡አሁን የሌላ የእናቶች ምናምን ምርመራ ከሌላው ሰው በፊት ነው ፤ቅድሚያ ተሰቷቸው ነው እሚመረመሩ፤ ሌሎች ከመመርመራቸው በፊት፡፡ ግን የቲቢ ታካሚ ተብሎ ለሄዱትም ቅድሚያ ቢሰጥ ጥሩ ነው፡፡እንዲያውም ወደ ሌሎች እንዳያስተላልፉ ቶሎ ብሎ ታክመው ቢወጡ ለጤና ጣቢያውም ጥሩ ነው ፡፡ወደ ሌሎች እንዳያስተላልፉ፤ እንደዛ ስለሆነ ያን ማድረግም ስላልተቻለ ፤ይህን ያህል ያው እሚታየውና እሚታከመውም ሰው የተለያየ ነው፡፡ምክንያቱም ብዛቱ ስታየው ያው ከኛ እንኳን አሁን እንደነገርኩህ አንድ ታካሚ ብቻ ነው ያለ፡፡ግን በትክክለኛው ቢመረመር ብዙ ታካሚ ይኖራል ትኩረት ተሰጥቶት ቢሰራበት ብዙ ታካሚ ይኖራል ፡፡እና እንደዛ ነው፡፡ያው ከመንግስት አንጻር እምጨምረው ስልጠና ካለመስጠቱም በተጨማሪ ደግሞ የተሰሩ ስራወችንም አለመገምገም፤የወደቀ ተግባርንም ደግሞ ግብረመልስ አለመስጠት ፤ያሉትን ነገሮች እነዚህ ተሰሩ እነዚህ አልተሰሩም ተብሎ ግብረ መልስ አለመስጠት አንዱ ችግር ነው፡፡ሌላው ደግሞ ከግብአት አኳያ ፤የላናቸው ሰወች ፎርም የለም፤ እንዲህ የለም ተብለው መመለስ ነገርም አለ፡፡እነዚህ እነዚህ ከግብአት አኳያም እሚቀር ነገር አለ፡፡ሌላው የሰው ሃይል የብቻውን ቲቢ ተጠርጣሪወችን እሚለይ ፎካል እራሱ አለመኖሩ አንድ ትልቅ ቻሌንጅ ነው፡፡ከዛ ውጭ ላብራቶሬ ሙያተኛም በቂ የሆነ ላብራቶሪ ሙያተኛ አለመኖር ፡፡ቢያንስ ወደታች ወርዶ ያሉትን እንትኖች አክታወች ወስዶ ወደዛ ለመመርመር እሚያበቃ ሙያተኛ አለመኖሩ፤ እነዚ ችግሮች ናቸው፡፡ ከዛ ውጭ የትራንሰፖርትም ችግር በጣም ሩቅ የሆኑ ቦታወችንና ደከም ያሉ ሰወችን አንቀሳቅሰን ለማምጣት እኛም ተንቀሳቅሰን ለመቀስቀስ የትራንስፖርት ችግር አለ፡፡እነዚህ ነገሮችም አንድ ለዚህ ለስራው መውደቅ ትልቅ እንትን ናቸው ፡፡በየጊዜው የቲቢ ስራወች አለመገምገም ፡፡እንደሌሎች ስራወች ታይተው በየጊዜው እነዚህ ስራወች አለመገምገም አንድ ትልቅ ችግር ነው፡፡ሌላው ወደ ሙያተኛው እምጨምረው ነገር ፤ስለ ቲቢ ታካሚወች ቤተሰብ ግንዛቤ አለመስጠት፡፡አሁን ግንዛቤ ቢኖራቸው እኛ እንኳን ተመርመሩ ባንላቸው እህቴ በሽተኛ ናት ወንድሜ በሽተኛ ነው የቲቢ እና መመርመር እኔ እፈልጋለው ማለት ይችል ነበር፡፡እንደሌላው ህመም፤አና እንደዛ ስለሆነ ግንዛቤ አለመስጠትም አንዱ ችግር ነው፡፡ከዛ ውጭ ቁርጠኝነትና ተነሳሽነት አለመኖር ትልቁ ቻሌንጅ ነው፡፡ሌላው ቅድም ከላብራቶሪ ሙያተኛ ላይ አንስቸዋለው፤የኛም ቢሆን በቂ ሙያተኛ የለም፡፡እኔ ብቻ ነኝ ከዚህ ተቋም ጤና ኬላ ያለሁትኝ እኔ ብቻ ነኝ፡፡አሁን ያለው ደግሞ የጠየና ኤክስቴንሽን ተግባር ከአስራ ስምንት በላይ ያሉ ተግባራት ናቸው ያሉ፡፡እነሱን ለማስፈጸም ኳሊቲውን ለማስጠበቅ የስራ ጫና አለ፡፡የስራ ጫና አንዱ ቻሌንጅ ነው፡፡ምክንያቱም ያው ስራ ሲበዛ መንጠባጠብ ይሆናል የሆነ አንዱን ይዘህ አንዱን መተው ነገር ስለሚኖር እነሱ ነገሮችም አንድ ችግር ናቸው፡፡ ሌላው ወደ በሽተኛውም ስንሄድ የቲብ በሽታ ካንዱ ወደ አንዱ እንደሚታላለፍ እውቅና አለመኖር ፡፡ቢያውቁም እንኳ ያን ያህል ስጋት እሚያስገባ ነገር አለ ብለው አለማሰብ፤የነሱም አንድ ይህ ቻሌንጅ ነው፡፡ከዛ ውጭ ከበሽታው በላይ ደግሞ ቅድሚያ ስራቸውን መስጠት፡ስራቸውን ቅድሚያ መስጠት ፡፡እነዚህ ነገሮች ስራወችን ወደ ኋላ ያደርገናል፡፡ ቲቢ በሽታ ምርመራ ንቁ ተሳትፎ አለማድረግ፡ ተነሳሽነት አለመኖር፡፡ያው ሳል ሳል ነው ዛሬ ያስለኛል ፤ነገ ይተወኛል አይነት ነገር እንጅ ፤የክፋ ይሰጠኛ ብሎ አለማሰብ ነገርም አለ፡፡እንደዛ ስለሆነ፡፡ሌላው ከማህበረሰብና ከኢኮኖሚያቸው አኳያ ሲታይ ፤ያው እንደ አገራችን ሁኔታ ሲታይ በኢኮኖሚም ዝቅ ያሉ ስለሆኑ ፤ወደዚህ ለመምጣ ትራንስፖርት አለ ምን አለ፤ከገጠር ወደ ከተማ እሚመጡበት ትራንስፖርት አይገኝም እንግዲህ፡፡አክታቸው ምናምን እሚሰራለቸው እሚስራለቸው ደግሞ ከጤና ጣቢያ ነው፡፡እና እራቅ ያለ ስለሆነ ከኢኮኖሚም አኳያ፤ ከኑሯቸውም ዝቅተኛ ኑሮ አኳያ ፤ይህም ቻሌንጅ ያደርጋል፡፡ከዛ ውጭ ደግሞ ያላቸውን ማህበራዊ ኑሮ መገለል አይነት ነገር፡፡ያው ከአንዱ ወደ አንዱ እንደሚተላለፍ ይታወቃል፡፡ግን ደግሞ በጣም ልክ እንደሌላ በሽታ አርጎ መቁጠርና ቲቢ አለበት የተባለን ሰው እንደሌላ ነገር ማግላል ይታያል፡፡እነዚህ ነገሮች ስላሉ ይህም አንዱ ችግር እሚፈጥርብን ነው፡፡ከዛ ውጭ ደግሞ ቤታቸው ሩቅ መሆኑ ፤ሁሎችንም ቤተሰብ አምጥቶ ለማስመርመር እራሱ ችግር አለ፡፡ከመመርመሪያ ቦታው እስከ ቤታቸው ያለው እርቀት በራሱ አንዱ ችግር ነው፡፡እሚል ነገር ነው ያለኝ እንጨርስ፡፡

ጠያቂ፤ በወረዳ ወይም በጤና ጣያ በኩል ስለ ቲቢ ታካሚ ቤተሰብ መመርመር የሚያደርጉላችሁ ድጋፍና ግብረ ምልስስ ምን ይመስላል

ኤክስ 3፤ያለው ነገር ያው መመርመር እንዳለባቸው ዝም ብሎ እንደዚሁ ይነገረናል፡፡ግን መመርመር አለባቸው ተብሎ፤ በደንብ ልክ እንደሌላው ተግባር፤ዛሬ እከሌ ቲቢ ተይዟል የከሌ በተሰቦች ዛሬውኑ መመርመር አለባቸው ወይም ምን ሲሆን መመርመር አለባቸው፤ እሚለውን በግልጽም አናውቀውም ስልጠናም ተሰቶንም አያውቅም፡፡ግምገማም አስመረመራችሁ አላስመረመራችሁ ግምገማ ላይም አይገባም ይህ፡፡እንደዛ ስለሆነ ይህ ያው አንዱ መንግስት ትኩረት አለመስጠቱን ያሳያል፡፡

ጠያቂ፤ ባህላዊ ህክምናና አስተሳሰብ ቤተብ እንዳይመረመር ተጽኖው ይኖር ይሆን

ኤክስ 4፤አለው እሱ ተጽኖ አለው፡፡እንደምታየው ግንዛቤ ካልተሰጣቸው ከባለሙያው ልቅ ጎረቤታቸውን እሚያምን ማህበረሰብ ነው ያለ፡፡እንደኛ እንደገጠር ማህበረሰብ አሁን አንተ መተህ ሙያተኛው ቲቢ እንደዚህ ነው ብለህ ከምትነግረው፤ማህበረሰቡን ነው እሚያምን፡፡ምክንያቱም አሁን ቅድምም ብየዋለሁ፤ የኛ ስልጠና ካልተሰጠን እነሱን ማሰልጠን አንችልም ፡፡አሁን አንተ ትንሽ ነገር ይዘህ መመርመር አለብህ እከሌ ቲቢ ስላለበት አንተም እንዳይተላለፍብህ ተመርመር ስትለው ለምን ነው እምመረመር ብሎ እንኳን ሲጠይቅህ ምላሽ አይኖርህም፡፡በደንብ ካላወቅኸው፤ እንደዛ ስለሆነ አለው ተጽኖ አለው፤እየሄዱም እሚወስዱ መድሃኒት አሉ፡፡የባህል ሃኪሞች በዛ አሉ ፡፡ስለዚህ ከዛ እሚጠቀሙ ብዙ በሽተኞች አሉ፡፡እንደዛ ስለሆነ አለው ተጽኖ አለው፡፡

ጠያቂ፤ ማጠቃለያነ..በመጨረሻ እሚጨምሩት ነገር ካለ፤

ኤክስ 4፤ያው ማለት እነዚህ የተናገርኳቸው ነገሮች ይህን ችግር ሊያበብሱት እሚችሉ ነገሮች ናቸው፡፡እነዚህ ችግር ሊያበብሱት እሚችሉ ነገሮች ናቸው ተብሎ ከታሰበ ይህ ጥናት ሲደረግ ለመለየትና እንትን ለማለት ስላልከኝ ቅድም ፤በትክክል ጥናቱ ታይቶ ችግሮች እሚለዩ ከሆነ ትኩረት ቢሰጠው የሙያተኛው ስልጠና ቢሰጠው፤ሙያተኛው ደግሞ በተሰጠው ስልጠና ልክ ደግሞ ለማህበረሰቡ ስልጠና እንዲሰጥና ለሚጠየቀው ጥያቄ ምላሽ ቢሰጥበት እሚል ነገር ነው ያለኝ፤ያለው ነገር ተጽእኖ አለው ፡፡በባህልም ሆነ በመንግስትም ሆነ በራሱ በሙያተኛውም በማህበረሰቡም ፡፡እንደዛ ስለሆነ ጠቅለል ያለ ልክ እንደ እናቶችና ህጻናት ፕሮግራም ተይዞለት ይህም ቢሆን ግብረ መልስ ቢሰጠው ፡፡በየጊዜው ግመገማ ቢደረግለት የተሻለ ነው ፡፡ይህ ነው ያለኝ ጨርሻለው፡፡

ጠያቂ፤ አመሰግናለው፡፡

**EX 4**

Questioner. Tell me about your experience.

X 4; There are many problems that prevent us from investigating of contacts. Of course, even though we don't know clearly that it will be investigated, we have a slight aware of being screening ccontacts; there are different problems for us to investigate them clearly. For example, when you see it from the government, we must take clear training to investigate contacts. In order to support and advice them; we must know the inside before. We have to wake them up. We can't wake them up or teach them when we are not given training. The society can be investigated in the form we have explained to them, but not just. And the attitude of the government about TB contacts is not that much. As you can see, the activities of mothers and children are not equal to the activities of TB. Because mothers are given priority. The woreda governments only said identify if there is TB suspect, but they don’t give training about what is TB and how contacts are screened seriously. Besides, by going to church; we said to the community if you have a cough, you should be examined, but we believe that there is no health extension, which knows what kind of cough the person has and we don’t tell them clearly due to lack of training. Because we have no aware. Because of that, the government is cold and does not pay attention to it, so we don't get much training. When they say they need to be examined, we tell them that you should be examined at the health center. But if we tell them that there is this this problem, people go.But since we are not awared enough, we say go and get checked, and they didn't go, they didn't get any check-up. Apart from that, there are some gaps on our side as well as on the professional side. Yes, those gaps on our side are they said, sent to the health center to examine the families of the sick, and since we don't know the problem and the harm, we don't send much contacts and we don't have an active participation to do that. Apart from that, when the contacts go to health center, not only the barrier is from the extension which we sent them by paying attention, but the health workers in the institution don’t paid attention to the ones we sent, they tell them once there once there to do the examination, and they come back by giving up with no screening when they complain, and this is because of the lack of attention from the experts and the health center. This describes the problem of the professional. Apart from that, it is not given priority. These TB patients and contacts are not given priority. Now, any examination of other mothers is done before anyone else. They are given priority to be examined, before others investigate. But it is good if priority is given to those who go as TB patients. In fact, it is good for the health center if they are treated quickly so that they do not spread it to others. Because it is like that and it is not possible to do that, the person who is seen and treated is different. Because when you look at the number of contacts and patients, even from us, as I told you now, there is only one patient. But if it is examined correctly, there will be many patients. If it is given attention and treated, there will be many patients. From the point of view of the government, in addition to not providing additional training, one of the problems is not evaluating the work done, not giving feedback on failed tasks, not giving feedback on the existing things that have been done or not. There is something to say that there is no such thing. Another thing is that in terms of resources, we don't have any form and anything for identified contacts people and come back with nothing done. There are things that need to be done in terms of resources. The other is lack of a focal person to identify TB suspects alone with no other job is a big challenge. Apart from that, there is a lack of enough laboratory professionals. The lack of a qualified professional to go to home of contacts and take sputum and examine it; these are the problems. Apart from that, there is also the problem of transportation to move people from very far places and bring tired people and to go to home of contacts. These things are one of the big reasons for the failure of the work. Not reviewing and evaluating the work ofTB works regularly. Not evaluating these cases regularly like other cases is a big problem. another thing I would like to add to the professional is not giving awareness about the families of TB patients. If they had awareness now, if we didn't even check them, contacts will say my sister is sick, my brother is sick with TB and I need to be checked like any other disease, lack of awareness is one of the problems. Apart from that, the lack of commitment and motivation is the biggest challenge. Another, as I explained before from lack of laboratory professionals and ours is not enough health extension workers. I am the only one who works in this health post. Currently, there are more than 18 tasks of extension. There is a workload to maintain the quality and to carry them out. Workload is one of the challenges. Because when there is work load; there will be dripping. They are also a problem because there is always something to take with you and leave something behind. Another thing is that when we go to the patient and contacts, there is no awareness that TB disease is transmitted from one person to another. Even if they know, not to think that there is something that poses such a threat and this is one of their challenges. Apart from that, they should give priority to their work over screening, to give priority to their work. Lack of active participation in TB diagnosis: lack of motivation.It holds us back. The same cough is a cough that bothers me today, but it will leave me tomorrow, there is something not to think that it will get worse. It is like that. the other is from the point of view of their social and economy, and the same as the situation of our country, since they are economically low, is there any transportation to come here, there is no transportation for them to come from the countryside to the city. Since their sputum is examined in the health center, In terms of their low standard of living, this creates a challenge.Apart from that, there is something like stigmatization of their social life. It is known that it is transmitted from one person to another. But it is also very similar to treating a person who has TB as another disease and ostracizing a person who is said to have TB as something else. Because these things exist, this creates a problem for us. Apart from that, the fact that their homes are far away, there is a problem in itself to bring all the families for testing. The distance from the testing place to their homes. It's a problem in itself. Let's finish what I have to say.

Probing; what is the support and feedback given to you by the district or health department about TB patient's family examination?

X 4: We are told that they should be investigated. But they should be investigated, well, just like any other activity, people who have been diagnosed with TB today should be their contacts tested today or they should be tested is We don't know what clearly, we have never been trained. Whether sent or not, is not included in the review meeting. It shows that the government is not paying attention.

Probing; will there be an impact on traditional medicine and thought not to be investigated?

X4; said, it has influenced. As you can see, if they are not given the awareness, there is a community that trusts their neighbor rather than the professional. Like our rural community, now if you say TB is transmitable and families shall investigated, that they don’t hear professional, tey believe the joke of the community. If we are not given our training, we will not be able to train them. Now, if you have to take a little something knowledge and say the community to get tested, Since Ekele has TB, you don't even get a response when they asks you to why to get tested, If you don't know it well; that’s why it has an impact there are traditional healers there, so there are many patients who use it.

Interviewer; finally, if you have anything to add;

X 4: That is, these things that I have said are things that can make this problem worse. Also, I would like him to provide training to the community and respond to the questions that are being asked. What he has has an impact, whether it is on the culture, the government, the professional himself or the community. Because that is the case, a comprehensive program like mothers and children should be given feedback. It is better if it is evaluated regularly. This is all I have.

Interviewer; Thank you.

**EX 1**

Extension 1; Together with their families. Except for monitoring these patients to take the same medicine, no examination was done with their families, because first of all, **there is no support from the district**. They should have been examined before, when the experts of the district or health center come for support, even if they come once or twice a year, it is another job. Pay attention. They never acknowledged the need to diagnose the problem with TB patients. We **have not received any training before**, so we did not have their families examined. Besides, it is said that there is **a lack of resource**s. Sometimes now we just learned; family same When we learned before, if a person's family is a pulmonary TB patient; It is said that it is the family that should be examined. Therefore, when we sometimes say take their families and examine them, others say that there is a lack of resources when these people are being examined. There is a lack of professionals. There is a lack of resources. It is said that there is a shortage of reagents, and since there is, we do not refer to them that much They refuse to go. They don't have it, because the symptoms are there but they don't have it, so they don't want to go. These two people were found after going to Bure Hospital. They came with the same medicine and were sent to the health center. From the health center to the health checkpoint, the medicine came. We gave them two months ago. From the health checkpoint, they have been taking the medicine every month for three months now, but their families have not been tested. There is a l**ack of manpower**, when we collect suspects and call them, they say that we do not have manpower, the labs are too small, we will not send them to them, and they will come here. No investigation has been done yet. They say there is a **lack of budget and transportation**. They say that the first thing to do is to leave the health center and the health post to do the investigation. Because the distance between the health center and the health post is far, it is very difficult to do the post because of the lack of budget and transportation services. They go to the health center to do the test and they have a lot of difficulty because there is a transportation problem. We have a lot of trouble to do the test. **It is because of the budget and transportation**. And it is for other work, but they don't pay much attention to the TB program. We did not pay attention to it. In the future, we should pay attention to them and create awareness. The other thing from the perspective of the patients and their families is that there is a **problem of awareness**. **There is a lack of awareness**. We did not make them aware of it, even if they were made aware of it, the problem is that they are **reluctant** to do it. They g**ive priority to their work**. The patients do not think that the TB disease from them will be transmitted to their families. But Emiayu, now they take their medicine as their priority because it doesn't make them sleep. Because the disease doesn't make them sleep, they do their work, **so they give priority to their work**. Now, one of the people is in a very **far place**, especially one patient who is in Mecha Dombar, and those people are now having a hard time leaving their families to get tested for TB. **The first transport is a motor and most of the motors are expensive**. It would be easier if someone else paid for that engine and tests. It would be possible if one person paid for that engine and tested. It would be very difficult for the entire family to come and do the test because **it is far away and has ups and downs**. And because it is far away, they don't want to do an investigation. We just taught them, and we made them aware. Another is that their **neighbors ostracize** them. They don't want to expose the disease because they think that. The reason is that if the whole family is examined, something else will happen; Inferior; They think that the society. So they are now left alone; to keep the secret; But if every family who wants to be investigated, the society will isolate us; They think because they are TB patients. Their families will pass it on to us; Because they say that they separate us because they think that; And they don't want to be kept secret, that's why they don't want to investigate. The transportation is also a problem, so we are asking them to come and do a screening here. We will ask him further

Probe; You have explained to me that it is a problem of transportation and distance, maybe you have made an effort to go to the people's house and investigate?

Extension 1: We did not make any effort. We did not make any effort to go and examine their families. The reason is that it is far from the Health post to reach people's homes, so there is no **transportation**. There is a motor. But the motor is difficult, the price is very expensive, and the road is not convenient.

Interviewer: lack of manpower and resources, do you have anything to ask the health center?

Extension 1: Lack of human resources and resources at the health center. Whenever we meet, we always have a meeting with the health center. When we meet, we raise the question. We always raise the question because there is no one and they say wait in line. They say we will fix it. We will always fix it later, and the input will come.

Questioner: In order to provide awareness, they may not be educated or read, so even if there is a distance, could you please explain to me if there is anything that you have given health education on your part, maybe the first work of health extension is going to the church or going to the place nearby? Extension 1: Now we are actually doing health extension work first to give health education. Awareness is creation. And we will create awareness, but mainly because the district and the health center did not pay attention to the creation of awareness. Because we tell them that if it is pulmonary TB, it is transmitted from one person to another through breathing. Always. And we didn't pay attention to the fact that they should be investigated. We create the same impression.

Questioner. Another idea to add or from If you have any questions that are not clear, please let me know

Extension 1: The context in which the question was asked is clear. But what I am giving you now is because I think that your university (the project) will prepare this question for the future. So, collect this worm now because it will lead to something good, so that it doesn't just stay here, tomorrow TB patients will be transmitted from person to person.

Questioner: Summarizing what has happened so far, please explain from the perspective of the district that support will be provided, but they have told me that no attention will be paid to TB. They told me that there is a lack of resources and a lack of human resources, for example, there is a problem with laboratory reagents and service at the health center. From the point of view of patients, there is a cognitive problem, putting their work first and not paying attention to the disease. In terms of the community or the area, they have revealed to me that there is a problem with the society's attitude so far, that they isolate patients and that patients face transportation problems due to the distance with their families.

Case 1: I don't have that much idea. Thank you for paying attention to the TB program. We will also do good things in the future.
