# Supplementary material for: Exploring barriers of household contact screening of index case contacts of pulmonary tuberculosis cases in Sekela district, Amhara region, Ethiopia: 2023; descriptive qualitative study
Source: PLoS One. 2025 Dec 30;20(12):e0339992. doi: 10.1371/journal.pone.0339992 (PMC12753078; doi:10.1371/journal.pone.0339992)
Supplement: Attachment — Submitted filename: point -by- point response.pdf [file pone.0339992.s004.pdf]

To **PLOS ONE** reviewer

We would like to appreciate your effort to made correction for this paper and here are my responses to the given comments

Reviewer: 1. The order of "Categories" in Table 2 should align with the order of terms (e.g. . Lack of Human power, Lack of adequate budget, Lack of training ...) are discussed in the text.

Response: we would like to say thank you and we made previously mistake on it. We saw these in detail and made correction. The orders and consistent use of words/ phrases in table 2 and discussed was made. Thank you again!!

2. Check typo carefully. For example "Land scape", "Tb officer"

Response : thank you for your insightful view, suggestion, comment and your time to see the typo problems in detailed; we made corrections of these and others in the manuscript.
3. This sentence "To ensure social representativeness,
a range of participants were involved, including the TB Woreda-level coordinator, focal persons, contact cases, and index cases" appeared twice.

Response: yes there was problem of repeating this sentences twice, we removed the repetition and made correction. Thank you very much!!
