# Supplementary material for: Exploring barriers of household contact screening of index case contacts of pulmonary tuberculosis cases in Sekela district, Amhara region, Ethiopia: 2023; descriptive qualitative study
Source: PLoS One. 2025 Dec 30;20(12):e0339992. doi: 10.1371/journal.pone.0339992 (PMC12753078; doi:10.1371/journal.pone.0339992)
Supplement: Attachment — Submitted filename: Response to Reviewers.docx [file pone.0339992.s003.docx]

This is the point by point response to the reviewers

Thank you dear reviewers, we have made revision based on your valuable comment. We learn a lot from your comment and if additional comment, we are delightful to revise again.

Here are the requested revisions

1. Please ensure that your manuscript meets PLOS ONE's style requirements, including those for file naming.

**Response**: we revised to comply with PLOS ONE’s formatting and file-naming requirements, following the provided style templates.

2. We noticed you have some minor occurrence of overlapping text with the following previous publication(s), which needs to be addressed:

**Response**: we have revised and paraphrased the manuscript

3. Please provide a complete Data Availability Statement in the submission form, ensuring you include all necessary access information or a reason for why you are unable to make your data freely accessible. If your research concerns only data provided within your submission, please write "All data are in the manuscript and/or supporting information files" as your Data Availability Statement.

**Response**: we have made correction on the system

4. When completing the data availability statement of the submission form, you indicated that you will make your data available on acceptance. We strongly recommend all authors decide on a data sharing plan before acceptance, as the process can be lengthy and hold up publication timelines. Please note that, though access restrictions are acceptable now, your entire data will need to be made freely accessible if your manuscript is accepted for publication. This policy applies to all data except where public deposition would breach compliance with the protocol approved by your research ethics board. If you are unable to adhere to our open data policy, please kindly revise your statement to explain your reasoning and we will seek the editor's input on an exemption. Please be assured that, once you have provided your new statement, the assessment of your exemption will not hold up the peer review process.

**Response**: we have made based on the recommendation

5. Your ethics statement should only appear in the Methods section of your manuscript. If your ethics statement is written in any section besides the Methods, please move it to the Methods section and delete it from any other section. Please ensure that your ethics statement is included in your manuscript, as the ethics statement entered into the online submission form will not be published alongside your manuscript.

**Response**: we have removed from the declaration section

6. If the reviewer comments include a recommendation to cite specific previously published works, please review and evaluate these publications to determine whether they are relevant and should be cited. There is no requirement to cite these works unless the editor has indicated otherwise.

**Response**: we have addressed based on their recommendations

Review Comments

Reviewer #1: This manuscript addresses an important topic related to tuberculosis (TB) contact investigation and barriers to effective implementation. The study provides valuable qualitative insights from multiple perspectives, including healthcare system, health workers, and patients. Overall, the structure of the Methods and Results sections is appropriate, and the thematic analysis approach is suitable for the research objectives.
However, several areas require clarification, consistency, and refinement to improve readability, methodological transparency, and the rigor of reporting. The following specific comments are provided to guide revision.
1. Section 3.1Study Area and Period
• Add a space in “96627(51%)” → should read “96,627 (51%).”

**Response:** we appreciate this comment and we made correction.

2. Section 3.3 –Study Participants and Sampling Procedure
• Define the abbreviation HEWs at first mention.

**Response:** thank you for this, we have first define it as health extension worker

• Clarify why redundancy was defined by responses from three people (“Redundancy was considered to occur when three people overlap in essence of their responses.”). Explain whether this threshold was based on prior qualitative literature or a team consensus.

• Suggested sentence revision for clarity and grammar:

“To ensure social representativeness, a range of participants were involved, including the TB Woreda-level coordinator, focal persons, contact cases, and index cases.” Clarify why redundancy was defined by responses from three people (“Redundancy was considered to occur when three people overlap in essence of their responses.”). Explain whether this threshold was based on prior qualitative literature or a team consensus.

**Response:** we corrected the grammar and accepted the given comment. For “Redundancy was considered to occur when three people overlap in essence of their responses”. Explain whether this threshold was based on prior qualitative literature or a team consensus.” We decide this as team consensus based on previous literature

**3. Section 3.5 – Data Collection Methods**

**• Define DHIS at first use.**

**• Ensure consistent line spacing throughout this section.**

**• Add space in “23- 42minutes” → should read “23–42 minutes.”**

**Response:** we accept the comment

**• The sentence “Notes were taken at each interview that used as backup file if there were lost or damaged records happened accidentally” needs clarification. Please indicate whether any data loss or damage occurred in this study.**

**Response:** there was no lost file in this study, we detail explained in the manuscript as Notes were taken during each interview and used as backup records in case of accidental data loss or damage; however, no data loss or damage occurred during this study.

**• The sentence “It mainly focused on collected data in the ??? and from field notes” appear incomplete — please insert the missing text.**

**Response:** we inserted the missed word in the document

Clarify how observations of facial expressions, reluctance, emphasis, and frustration were incorporated into data interpretation, as these aspects are not reflected in the Results section.

**Response:** we appreciated this helpful suggestion and we have made correction as. Nonverbal cues, including facial expressions, tone, and signs of hesitation or frustration, were noted to contextualize responses and guide interpretation, though not all are reported verbatim in the Results.

• Specify how many times each transcript was read during thematic analysis (“Transcripts were read and reread…”).

**Response:** we have made revision based on this concept

**4. Section 3.8 – Trustworthiness and Quality Assurance**

**• Correct typographical errors:**

**o “audit trial” → audit trail**

**o “confirm ability” → confirmability**

**c** we have accepted this suggestion and made revision, thank you very much!!

**• Briefly define member checking and confirmability for readers unfamiliar with these qualitative terms.**

**Response:** We briefly explained this on the manuscript in bracket

**• Specify how many individuals participated in the peer-review process for data analysis and interpretation (“The data analysis, interpretations, and conclusions were continuously peer reviewed…”).**

**Response:** thank you we add three colleagues were participated in peer review

5. Participant Characteristics (Table 1)

• Clarify the educational levels described (avoid abbreviations such as “level 4”). Write them out (e.g., “secondary education,” “college diploma,” etc.).

**Response:** thank you very much we have made correction

• Correct “where as” → whereas.

• Verify missing information for participant C1 (education, marital status, residence, occupation).

**Response:** yes we missed and we add the missed

• Clarify the meaning of “Phase of RX of Ps”—what do “RX” and “Ps” represent?

**Response:** Rx=treatment and pts=patients we fully explained in the document

• Remove the religion column if all participants are Christian.

• Consider combining the last two columns into one “Notes” column for simplicity.

**Response:** Since merging and labeling “Notes” may confuse readers we merged and replaced with phase of treatment and number of contacts respectively

• Avoid abbreviations for participant codes.

**Response:** thank you we avoided the abbreviations and wrote in full form

6. Thematic Findings (Table 2 and Results Section)

• Explain the meaning of “not reviewing” under the subtheme “Health sector–related barriers.”

**Response:** thank you it is to mean not reviewing mean not undertaking review meeting

• Please check the consistency between table 2 and results for each category. You should verify that

o Every subtheme and illustrative quote in Table 2 appears and is discussed in the Results section.

**Response:** thank you very much we made correction; there were ordering problem in the table and result section

o The wording of categories and subthemes matches exactly (e.g., capitalization, phrasing).

**Response:** we separteded themes and subthemes

o The order of presentation in Table 2 aligns with how themes are described in the text.

• Correct page numbering starting from the page containing Table 2.

**Response:** We revised based on the comment, thank you

• Revise sentence for clarity:

“If the necessary conditions are not fulfilled by the government, performing contact tracing becomes very difficult.”

**Response:** we appreciated the comment we took it

• Correct minor typographical issues:

o “This results as ignorance of Tb contact screening.” → “This results in neglect of TB contact screening”

**Response:** we have great for such type of insightful review, we took it

o “house hold” → household

o “man power” → manpower

o “Up dated” → updated

**Response:** we corrected these typographical issues:

• Format improvements:

o Bold subtheme heading: “Lack of commitment” under Theme 2.

o Bold full theme headings:

**Response:** we have made correction on it

 Theme 3: Patient / Index Case and Contact–Related Barriers

 Theme 4: Social, Cultural, and Economic–Related Barriers to Contact Screening

**Response:** we have great appreciation for this valuable comment; we have made correction based on the recommended suggestions.
